# Effect of Particle Size on the Physicochemical and Functional Properties of Sweet Lupin (*Lupinus angustifolius*) Fermented with *Rhizopus oligosporus*

**DOI:** 10.3390/foods15183306

**Published:** 2026-09-18

**Authors:** Ihlana Nairfana, Jaspreet Singh, Mike Boland, Lovedeep Kaur

**Affiliations:** 1 School of Food Technology and Natural Sciences, Massey University, Palmerston North 4442, New Zealand; i.nairfana@massey.ac.nz (I.N.); j.x.singh@massey.ac.nz (J.S.); 2 Riddet Institute, Massey University, Palmerston North 4442, New Zealand; m.boland@massey.ac.nz

**Keywords:** *Lupinus angustifolius*, *Rhizopus oligosporus*, particle size, protein functionality, plant-based ingredients

## Abstract

Particle size influences microbial accessibility during solid-state fermentation and may therefore determine the extent of structural modification and functionality of plant-based ingredients. This study investigated how the particle size of lupin substrates prior to fermentation with *Rhizopus oligosporus* (*R. oligosporus*) influences fermentation behaviour and the properties of the resulting fermented flour. Split, crushed, and fine lupin fractions were fermented, freeze-dried, milled to flour, and compared with unfermented lupin flour. Fermentation modified the chemical composition by reducing fat content and altering dietary fibre composition through a decrease in insoluble dietary fibre and an increase in soluble dietary fibre, particularly in the crushed fraction. Amino acid composition was also influenced by fermentation, with higher total essential amino acid contents observed in the crushed and fine fractions than in the split fraction. Functional properties were strongly affected by particle size, with the fermented crushed fraction exhibiting the highest water holding capacity, protein solubility, emulsifying activity, foaming capacity, and viscoelastic strength. FTIR and differential scanning calorimetry demonstrated that fermentation induced controlled protein restructuring and molecular reorganisation without extensive denaturation, with the crushed fraction showing the most favourable balance between structural modification and protein stability. Multivariate analysis further confirmed that functional performance was governed by the interaction of compositional and structural changes. Overall, particle size is a critical processing parameter that regulates fermentation efficiency and determines the quality of fermented lupin flour.

## 1. Introduction

Lupins are gaining popularity as an excellent plant-based protein source for human consumption [1]. In New Zealand, food-grade lupin is predominantly imported from Australia as sweet *Lupinus angustifolius* (*L. angustifolius*), characterised by low quinolizidine alkaloid content [2]. These are commonly available as split beans and whole flour, which can be incorporated into a variety of food products, including breads, pasta, and noodles. However, lupin flour, like other plant-based food ingredients, is not composed solely of protein, but also contains starches, fibre, lipids, phenolic compounds, and polysaccharides, all of which influence their functional performance [3]. As a result, commercially available plant proteins are often highly refined, such as isolates and concentrates. While these refined ingredients offer advantages in purity and functionality, they are increasingly criticised for being overly processed or artificial [4]. Consequently, there is growing interest in less refined protein ingredients that can deliver comparable or even enhanced functionality, shifting the focus from purity to functionality [5,6].

Fermentation is a traditional and effective process used to modify the properties of plant-based ingredients. During fermentation, microorganisms produce enzymes that degrade complex macromolecules into smaller units, thereby enhancing nutritional, functional, and sensory attributes [7]. *R. oligosporus* is a well-known fungus widely used in the production of tempeh, a traditional fermented soybean product originating from Indonesia [8]. It is an edible filamentous fungus with a long history of use in the production of tempeh, a traditional fermented legume-based food, supporting its established suitability for food fermentation. In the present study, fermentation was conducted using the commercial food-grade tempeh starter RAPRIMA [9,10,11]. *R. oligosporus* is traditionally associated with soybean tempeh; however, its application has also been extended to other plant-based substrates. Previous studies have reported the successful production of tempeh from chickpeas [12], lentils [13], and different bean varieties, as well as cereal substrates such as barley [14]. Beyond its traditional application in tempeh, *R. oligosporus* has also attracted interest as a source of edible fungal biomass for food applications [15]. Its mycelial biomass has been investigated as a protein-rich food ingredient. Together, these applications show that *R. oligosporus* can be used to ferment a range of plant-based substrates. Although tempeh has been extensively studied, there remains a need for further optimisation, particularly under controlled conditions such as bioreactors, where parameters including temperature, pH, agitation, and oxygen availability can be precisely regulated.

Milling of lupin is an important pre-processing step to improve fermentability. To date, tempeh production has primarily utilised dehulled split beans [16,17]. Exploring different particle sizes of lupin offers the potential to better understand their influence on fermentability and overall fermentation outcomes. Smaller particle sizes increase surface area, thereby enhancing microbial access and activity [18]. Previous research has investigated lupin fermentation with *R. oligosporus* in relation to sensory quality, reporting that particle sizes below 5 mm resulted in inferior firmness and aroma [19]. However, limited information is available regarding the impact of particle size on the functional, nutritional, and thermal properties of fermented lupin. Therefore, this study aims to investigate the effect of different lupin particle sizes on fermentation with *R. oligosporus*, using a range of analytical methods to provide a deeper understanding of its overall fermentability and resulting properties. In this work, a commercially available food-grade lupin ingredient (marketed under the brand LupinQ) was selected, as to the best of our knowledge, the only edible lupin product currently available in the New Zealand market for food applications. This ingredient was chosen as it complies with Food Standards Australia New Zealand requirements for edible lupin products, including acceptable quinolizidine alkaloid levels for human consumption.

## 2. Materials and Methods

### 2.1. Materials

Food-grade split lupin beans (*L. angustifolius*) were purchased from Lupin Co. (New South Wales, Australia) marketed with the brand LupinQ with moisture content of 9–10% (Lupin Co., New South Wales, Australia), grown under a proprietary breeding program, and sourced from a single origin. Tempeh starter RAPRIMA, Bandung, Indonesia (contains *R. oligosporus*) was purchased from Nusantara Trading (Auckland, New Zealand). Unless otherwise stated, all chemicals and reagents were of analytical grade.

### 2.2. Milling Lupin to Different Particle Sizes

Three different bean particle sizes used in this experiment (split, crushed, and fine) were prepared using the dry milling technique. Dehulled split beans purchased from the supplier milled using a pilot-scale 15B series universal impact mill (Grain Tech Engineering Ltd., Auckland, New Zealand) and were passed through a 2.0 mm sieve (Glenammer Sieves, Ayrshire, UK) to obtain crushed and 1.0 mm sieve (Glenammer Sieves, Ayrshire, UK) to obtain fine particle sizes. These beans were then stored in a cool, dry room at a temperature of 20 °C until further processing.

### 2.3. Controlled Fermentation

Fermentation was conducted in a controlled bioreactor (BioFlo/Celligen^®^ 115, Eppendorf, NB, Canada) with temperature, pH, aeration and agitation maintained throughout the fermentation period. Lupin substrates with different initial particle sizes (Section 2.2) were weighed (420 g), mixed with 840 mL of water and boiled for 20 min. The cooked substrates were drained and air-dried at room temperature (20–22 °C) for 15 min until they reached room temperature. The cooked weight was recorded before inoculation. The substrates were then inoculated with 1% (*w*/*w*) commercial tempeh starter (RAPRIMA, Indonesia), a commercial food-grade starter used for tempeh production and reported to contain *R. oligosporus* as the principal fermentation microorganism [9,10,11]. Inoculated lupin substrates were mixed with 1 L of MiliQ water, and fermented for 36 h at 30 °C, 100 rpm and 1 L/min aeration. The same inoculum level and fermentation conditions were applied to all particle-size treatments.

### 2.4. Particle Size Distribution Before and After Fermentation

Particle size distribution of lupin fractions was determined both before and after fermentation by digital image analysis using ImageJ software version 1.54t (National Institutes of Health, Bethesda, MD, USA) according to Mazzoli et al. [20]. Three independent images were captured for each lupin fraction using a smartphone camera under controlled imaging conditions. Samples were dry dispersed as a thin layer on a matte white background to minimise particle overlapping. Images were captured using the same lighting conditions, background, camera orientation, and distance between the camera and sample surface. Due to differences in particle size, the number of particles captured per image varied among fractions, with approximately 15–30 particles per image for the split fraction, 40–70 for the crushed fraction, and 80–150 for the fine fraction, corresponding to approximately 100, 250, and 550 particles analysed per fraction, respectively. A ruler included within each image was used for spatial calibration in ImageJ. Images were converted to binary format, and thresholding was applied to separate particles from the background. Individual particles were identified using the “Analyze Particles” function, and Feret diameter was selected as the representative particle size parameter due to the irregular morphology of milled lupin particles. For each image, particle size distributions were generated from individual Feret diameter measurements. The value of Feret’s diameter (d_F_) is the longest distance between any two points along the selection boundary, also known as maximum caliper. Results are expressed as mean ± standard deviation from three independent image replicates.

Following completion of fermentation, all fermented samples were freeze-dried for subsequent physicochemical and functional analyses, and then milled and sieved to pass through a 1 mm sieve (Grain Tech Engineering Ltd., Auckland, New Zealand; Glenammer Sieves, Ayrshire, UK). The same freeze-drying, milling, and sieving procedure was applied to the unfermented control sample. Particle-size distribution was additionally determined using a Mastersizer 3000 (Malvern Panalytical, Malvern, UK) equipped with an Aero S dry dispersion unit, following the method described by Fan et al. [21]. The instrument has a measurement range of 0.01–3500 µm, with particle refractive and absorption indices of 1.53 and 0.001, respectively. Samples were introduced using the Aero S dry dispersion unit with the General-Purpose-Tray operated at a feed rate of 60%, a hopper gap of 1.5 mm, and a pressure of 3 bar on the standard Venturi dispenser. The particle-size distribution was characterised using the D10, D50, and D90 values.

### 2.5. Dry Matter Content and Dry Matter Recovery

All measurements of Dry Matter (DM) and dry matter recovery were performed using three independent fermentation batches, following published methods by Wang et al., [22]. DM content was determined gravimetrically using freeze-dried fermentation samples. At each sampling time (0, 12, 24, and 36 h), a representative wet sample was weighed, recorded as the wet sample mass, and subsequently freeze-dried to constant mass. The freeze-dried sample was weighed and DM content was calculated as:

DM (%) = (W_FD_/W_wet_) × 100

where W_FD_ is the mass of the freeze-dried sample (g) and W_wet_ is the mass of the corresponding wet sample collected during fermentation (g). Dry matter recovery (DMR) was calculated using a dry-matter mass balance, considering the initial dry lupin introduced into the fermentation system and the dry matter recovered from the fermented material. DM recovery was expressed as the percentage of initial dry matter retained following fermentation:

DMR (%) = (DM_final_/DM_initial_) × 100

where DM_initial_ represents the initial dry matter introduced into the fermentation system and DM_final_ represents the total dry matter remaining at the end of fermentation based on the measured DM content and corresponding fermentation mass.

### 2.6. Measurements of Physicochemical Properties of Unfermented and Fermented Lupin Flour

#### 2.6.1. Measurements of Proximate Composition

The crude protein of all lupin flour samples was determined according to AOAC 968.06 [23] following Dumas method using the nitrogen to protein conversion factor of 5.7. The moisture content was measured according to AOAC 930.15 [24]. Ash content was measured according to AOAC 942.05 [25]. Fat content was measured by the Mojonnier method according to AOAC 922.06 [26]. The starch content was measured according to AOAC 996.11 [27]. The total dietary fibre was measured by AOAC 991.43 [28]. Total carbohydrate content was calculated by difference (CHO % = 100 − (MC % + CP % + Fat % + Ash %)). All values are expressed as percentages (%).

#### 2.6.2. Measurements of Amino Acid Profiling

Quantitative determination of total amino acids according to AOAC 994.12 [29] using HCl hydrolysis followed by RP HPLC separation using AccQ tag derivatisation. Quantification of cysteine and methionine was performed according to AOAC 985.28 [30] through performic acid oxidation.

#### 2.6.3. Colour Measurements

Colour measurements of lupin flour were performed using a Minolta colourimeter (Chroma Meter CR-400, Hong Kong, China) operated in reflectance mode. The instrument was calibrated with a standard white reference tile prior to analysis. Colour parameters (L*, a*, and b*) were recorded using a D65 illuminant, a 10° standard observer, and an 8 mm measurement aperture. All measurements were conducted in triplicate. ΔE was calculated relative to the unfermented lupin flour using the CIELAB colour-difference equation as previously reported by Mao et al. [31]:

$$∆E_{\left\{ab\right\}}^{*}=\surd \left\{{\left(L_{2}^{*}-L_{1}^{*}\right)}^{2}+{\left(a_{2}^{*}-a_{1}^{*}\right)}^{2}+{\left(b_{2}^{*}-b_{1}^{*}\right)}^{2}\right\}$$

#### 2.6.4. Fourier Transform Infrared Spectroscopy Analysis

Fourier transform infrared (FTIR) spectroscopy was done to characterise the secondary structure of lupin proteins, following a method described by Gawat et al. [31]. Spectra of whole lupin flour were recorded using a Perkin Elmer Spectrum 2 FTIR instrument equipped with a diamond crystal universal attenuated total reflectance (UATR) accessory (Perkin Elmer, Shelton, CT, USA). Dried samples were placed directly on the diamond crystal and pressed gently using the instrument’s pressure knob and shoe press. Spectral data were collected across the mid-infrared range (400–4000 cm^−1^) with a resolution of 4 cm^−1^ and 32 scans per sample. Measurements were performed in triplicate and averaged. Background subtraction, baseline correction, and normalisation were performed using OriginLab Software version 2023 (Northampton, MA, USA). The spectra were interpreted across the entire spectral range to identify changes in characteristic functional groups associated with proteins, lipids, carbohydrates, and other molecular components. Protein secondary structure was further evaluated by deconvolution of the amide I region (1600–1700 cm^−1^) using Gaussian curve fitting. The relative proportions of β-sheets, random coils, α-helices, β-turns, and antiparallel β-sheets were calculated from the fitted peak areas according to established methods by Gawat et al. [32] where aggregated strands (1610–1620 cm^−1^), β-sheets (1620–1640 cm^−1^), random coils (1643–1650 cm^−1^), α-helices (1650–1660 cm^−1^), and β-turns (1660–1695 cm^−1^). Three independent fermentation batches were analysed for each treatment (*n* = 3), and the results of the secondary-structure analysis are expressed as mean ± SD.

#### 2.6.5. Differential Scanning Calorimetry Analysis

Differential scanning calorimetry (DSC) analysis was carried out using a TA DSCTM 2000 instrument (TA Instruments, New Castle, DE, USA) following a method described by Devkota et al. [33]. Approximately 10 mg of a 40% (*w*/*v*) lupin flour sample was placed in a hermetically sealed pan (TZero 901684.901, Red Thermo, Changsha, China). Samples were subjected to a heating and cooling cycle between 25 and 120 °C at a rate of 5 °C/min. An empty pan served as the reference, and the instrument was calibrated prior to measurements. Thermal properties of the flour were analysed using TA Universal Analysis software version 4.5A (TA Instruments, New Castle, DE, USA). The thermal denaturation temperature (Td) was identified from the peak of the endothermic transition, and the enthalpy change (ΔH) was calculated from the area under the peak.

### 2.7. Functional Properties of Unfermented and Fermented Lupin Flour

#### 2.7.1. Measurements of Water and Oil Holding Capacities

Water holding capacity (WHC) and oil holding capacity (OHC) of lupin flour were measured using the centrifugal method described by Beuchat cited in Barac et al. [34]. Briefly, 1 g of sample was combined with 10 mL of distilled water or canola oil (Pams, Auckland, New Zealand) and vortexed for 30 s. The mixtures were then left to equilibrate at ambient temperature for 30 min before centrifugation at 5000× *g* for 30 min (DYNAC II Centrifuge, Clay Adams, Franklin Lakes, NJ, USA). The volume of the separated supernatant was recorded using a 10 mL graduated cylinder. WHC and OHC values were calculated on a dry weight basis.

#### 2.7.2. Measurements of Least Gelation Concentration

Lupin flour suspensions were prepared in deionised water at concentrations ranging from 2% to 20% (*w*/*v*), increasing in 2% intervals, in test tubes as previously described by Chukwuejim et al. [35]. The samples were heated in a water bath (BUCHI Water Bath B-480, Flawil, Switzerland) at 80 °C for 1 h, then immediately cooled under running cold water. The tubes were subsequently stored at 4 °C for 2 h. The least gelation concentration (LGC) was identified as the lowest flour concentration at which the gel formed remained intact and did not flow upon tube inversion.

#### 2.7.3. Measurements of Emulsifying Activity and Stability

Emulsifying activity (EA) and emulsion stability (ES) were assessed according to a previously reported method by Rajpurohit et al. [36]. A 1% (*w*/*v*) lupin flour dispersion was prepared in distilled water, and the pH was adjusted to 2, 4, 6, or 8 using 0.1 M HCl or 0.1 M NaOH to examine the influence of pH on emulsifying performance. The dispersion was homogenised for 10 min using a high-speed homogeniser (Polytron PCU-2, Malters, Switzerland). During homogenisation, 100 mL of canola oil (Pams, Auckland, New Zealand) was slowly incorporated into the mixture at the fifth minute under continuous stirring. The resulting emulsions were transferred into graduated centrifuge tubes and centrifuged at 2000× *g* for 10 min (DYNAC II Centrifuge, Clay Adams, Franklin Lakes, NJ, USA). Emulsifying activity (%) was calculated as the ratio of the height of the emulsified layer to the total height of the mixture. Emulsion stability (%) was determined using the same emulsions prepared for EA analysis. The samples were heated at 80 °C for 30 min in a water bath (BUCHI Water Bath B-480, Flawil, Switzerland), cooled under running tap water for 15 min, and subsequently centrifuged at 2000× *g* for 15 min.

#### 2.7.4. Measurements of Foaming Activity and Stability

Foaming activity (FA) and foam stability (FS) were evaluated following a previously described procedure by Rajpurohit et al. [36]. A 1% (*w*/*v*) lupin flour suspension was prepared in distilled water, and the pH was adjusted to 2, 4, 6, or 8 using 0.1 M HCl or 0.1 M NaOH. Each suspension was whipped individually using a hand mixer (Breville, Sydney, Australia) at high speed for 5 min. The whipped mixture was immediately transferred into a 1000 mL graduated cylinder, and the foam volume was recorded. Foaming activity (%) was calculated as the ratio of foam volume to the initial total suspension volume. Foam stability (%) was determined by measuring the foam volume remaining after the samples were left to stand at room temperature for 2 h and expressing this value relative to the initial foam volume.

#### 2.7.5. Measurements of Protein Solubility

Protein solubility of the flours was evaluated using a previously reported procedure by Barac et al. [34]. Lupin flour suspensions (2%, *w*/*w*) were prepared in reverse osmosis (RO) water, and the pH was adjusted to 2, 4, 6, or 8 using 0.1 M HCl or 0.1 M NaOH. The suspensions were stirred at moderate speed for 1 h to facilitate protein dispersion. Following mixing, samples were centrifuged (RC6+ Centrifuge, Thermo Scientific, Osterode am Harz, Germany) at 17,000× *g* for 30 min to separate insoluble material. The resulting supernatants were freeze-dried, and their protein content was determined by the Kjeldahl method (AOAC 979.09) [37], using a nitrogen-to-protein conversion factor of 5.7. Protein solubility was expressed as the proportion of protein recovered in the supernatant relative to the total protein content of the original flour.

#### 2.7.6. Measurements of Rheological Properties

Dynamic rheological behaviour was evaluated using strain, temperature, and frequency sweep tests based on a previously reported method by Mao et al. [31], with minor modifications. The viscoelastic parameters measured included storage modulus (G′), loss modulus (G″), and loss tangent (tan δ). Flour pastes were prepared at a moisture content of 25% (*w*/*w*) and analysed using a controlled-stress rheometer (Anton Paar Physica MCR301, Graz, Austria) equipped with a 40 mm parallel steel plate (PP40) and a plate gap of 2 mm. Prior to temperature and frequency sweep measurements, strain sweep tests were performed at 25 °C over a strain range of 0.001–100% to identify the linear viscoelastic region (LVR) and to ensure that structural responses were not influenced by excessive mechanical deformation. A strain amplitude of 0.01%, which fell within the LVR, was subsequently selected for all temperature and frequency sweep experiments. Temperature sweep measurements were conducted to assess changes in viscoelastic properties during heating and cooling. Samples were heated from 25 °C to 95 °C at a rate of 4 °C min^−1^, held at 95 °C for 30 s, and then cooled back to 25 °C at the same rate, followed by a final holding period of 30 s at 25 °C. To reduce moisture loss during testing, a thin layer of mineral oil (Bio-Rad Laboratories, Hercules, CA, USA) was applied around the sample edges. Following the temperature sweep, oscillatory frequency sweep tests were carried out to evaluate the strength of the protein gel network. Measurements were performed at 25 °C over an angular frequency range of 0.01 to 10 Hz.

### 2.8. Multivariate and Pearson Correlation Analysis

Pearson correlation coefficients for relationships between different physicochemical and functional properties were calculated to provide an integrated assessment of the relationships among particle size, fermentation treatment, and the physicochemical, structural, and functional characteristics of the lupin samples. Variables were selected based on their relevance to the main objectives of the study and their ability to represent distinct aspects of lupin functionality and structure. The selected variables comprised protein content, starch content, low-gelation concentration (LGC), water-holding capacity (WHC), protein solubility, foaming stability (FS), β-sheet content, and denaturation temperature (Td). These variables were selected to provide complementary information on composition, hydration-related functionality, protein behaviour, and structural characteristics, while avoiding the inclusion of closely related measurements that could provide overlapping information.

Prior to PCA, the selected variables were standardised to account for differences in measurement units and numerical ranges. PCA was then performed on the standardised dataset, in which the original variables were transformed into a smaller number of principal components. Each principal component represents a linear combination of the standardised variables, with PC1 explaining the greatest proportion of variation in the dataset and PC2 explaining the next greatest proportion. The PCA scores were used to visualise the distribution of the samples according to their overall multivariate characteristics, while the loading values were used to identify the variables contributing to the separation along each principal component. The PCA was performed using SPSS software (version 17.0).

### 2.9. Statistical Analysis

All experiments were conducted with at least three replicates, and results are expressed as mean values plus standard deviation. Data was subjected to two-way analysis of variance for particle size before and after fermentation, otherwise other data were subjected to one-way analysis of variance (ANOVA), with mean comparisons performed using Tukey’s post hoc test at a significance level of *p* ≤ 0.05. Statistical analyses were carried out using SPSS software (version 17.0).

## 3. Results

### 3.1. Particle Size Distribution

**Figure 1:**
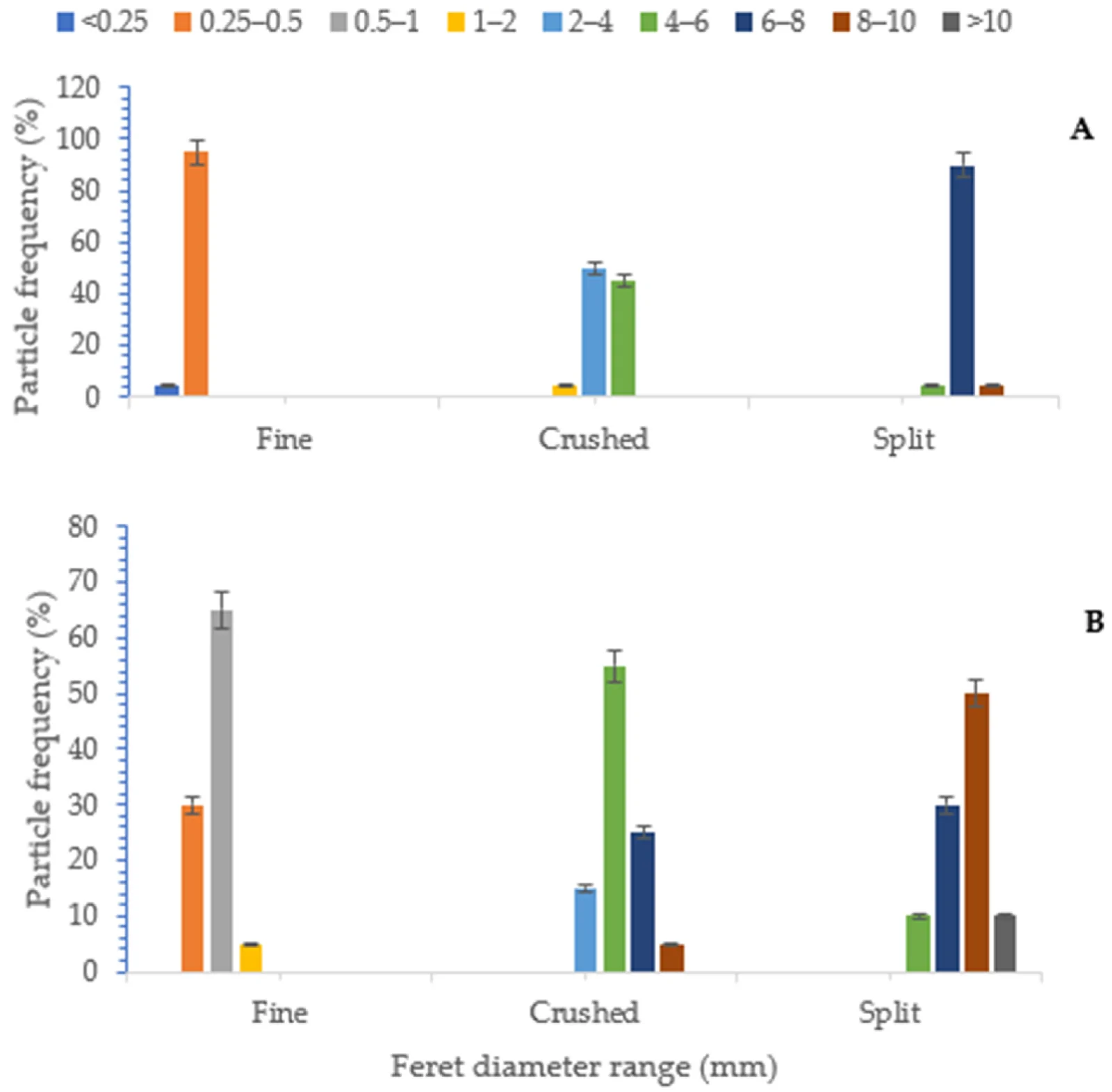
Particle size distribution (means and standard deviations) before (**A**) and after (**B**) fermentation considering shape factor d_F_.

Digital image analysis confirmed distinct differences in particle size among the three lupin fractions produced by different milling treatments (Table 1), and the percentage of particles count in each size range based on their dimensional parameters is shown in Figure 1.

Image analysis using Feret diameter demonstrated that the milling process successfully generated three distinct particle size fractions. The split fraction exhibited the largest mean Feret diameter (7.02 ± 1.60 mm), followed by the crushed fraction (3.72 ± 0.56 mm), while the fine fraction had the smallest average particle size (0.37 ± 0.25 mm). A similar trend was observed for the mean minimum Feret diameter, which decreased from 6.21 ± 1.04 mm for the split fraction to 2.92 ± 0.92 mm for the crushed fraction and 0.15 ± 0.07 mm for the fine fraction. These results confirm the progressive reduction in particle dimensions achieved through milling and indicate that the selected milling conditions effectively produced well-defined particle size fractions. Following 36 h of fermentation, the same relative ranking was maintained, with the split fraction exhibiting the largest mean Feret diameter (8.50 ± 1.72 mm), followed by the crushed (5.10 ± 1.21 mm) and fine (0.61 ± 0.09 mm) fractions. Similarly, the minimum Feret diameter was highest for the split fraction (7.20 ± 2.84 mm), followed by the crushed (4.41 ± 1.02 mm) and fine (0.27 ± 0.07 mm) fractions. Although the mean Feret and minimum Feret diameters were numerically higher at the end of fermentation than at the start, these changes were not statistically significant within the individual fractions (*p* > 0.05). Thus, fermentation did not significantly alter the measured particle dimensions, and the distinct particle-size characteristics established by the milling treatments were largely retained throughout fermentation.

The difference between Feret and minimum Feret diameters across all fractions further indicated the irregular morphology of the lupin particles. The minimum Feret diameter was consistently lower than the corresponding Feret diameter, reflecting differences between the longest and shortest caliper dimensions of the particles. This is consistent with the irregular geometry expected from mechanically fractured lupin cotyledons. The crushed fraction showed intermediate particle dimensions and relatively greater variability, consistent with a heterogeneous population containing particles of different degrees of fragmentation. In contrast, the split fraction retained the largest particle dimensions, whereas the fine fraction consisted predominantly of smaller particles.

Before fermentation (Figure 1A), the fine fraction was dominated by particles in the 0.25–0.50 mm range, accounting for approximately 95% of the measured particles when combined with the <0.25 mm fraction. The crushed fraction was predominantly distributed between 2 and 6 mm, with approximately 58% and 42% of particles occurring in the 2–4 and 4–6 mm ranges, respectively. The split fraction was dominated by particles in the 6–8 mm range, accounting for approximately 89%, with only small proportions occurring in the 4–6 and 8–10 mm ranges. The particle-size distribution (Figure 1B) also changed following 36 h of fermentation. The fine fraction showed a clear shift from being predominantly distributed in the 0.25–0.50 mm range before fermentation to the 0.5–1.00 mm range after fermentation, which accounted for approximately 64% of the particles, while approximately 30% remained within the 0.25–0.50 mm range and only about 5% were between 1 and 2 mm. The crushed fraction also shifted towards larger particle-size classes after fermentation, with 15% of particles distributed within the 2–4 mm range, 55% within the 4–6 mm range, 25% within the 6–8 mm range, and 5% within the 8–10 mm range. The split fraction exhibited a more pronounced shift towards larger particles, with 10% of particles in the 4–6 mm range, 30% in the 6–8 mm range, 50% in the 8–10 mm range, and a further 10% exceeding 10 mm. Thus, fermentation was associated with a shift towards larger apparent particle-size classes across all three fractions. However, despite these apparent changes in particle-size distribution, the differences in mean Feret diameter before and after fermentation were not statistically significant (*p* > 0.05).

### 3.2. Dry Matter Content and Recovery During Fermentation

Dry matter (DM) content varied among the particle-size fractions throughout fermentation (Supplementary Materials). The split fraction had DM contents of 24.01 ± 0.55%, 24.90 ± 1.43%, 24.48 ± 0.80%, and 23.61 ± 0.91% at 0, 12, 24, and 36 h, respectively. The crushed fraction showed a gradual decrease in DM content from 24.24 ± 0.30% at 0 h to 22.58 ± 1.62% at 24 h, followed by an increase to 23.61 ± 1.26% at 36 h. The fine fraction showed a similar overall decrease, from 23.22 ± 0.49% at 0 h to 21.88 ± 1.41% at 36 h. Repeated-measures ANOVA showed that fermentation time did not significantly affect DM content (F = 2.090, *p* = 0.137), and there was no significant time × particle-size fraction interaction (F = 0.910, *p* = 0.510). However, a significant main effect of particle-size fraction was observed (F = 11.933, *p* = 0.008, partial η^2^ = 0.799). The mean DM content across sampling times was highest for the split fraction (24.25 ± 0.15%), followed by the crushed fraction (23.59 ± 0.53%), while the fine fraction had the lowest mean DM content (22.86 ± 0.20%). Tukey’s post-hoc comparison showed that the split fraction had significantly higher DM content than the fine fraction (*p* = 0.008), whereas the differences between split and crushed (*p* = 0.181) and crushed and fine (*p* = 0.126) were not significant.

DM recovery remained high across all particle-size fractions (Supplementary Materials). The split fraction showed the highest recovery (97.20 ± 0.84%), followed by the crushed (95.52 ± 1.30%) and fine (94.53 ± 1.37%) fractions. Although a numerical decrease in DM recovery was observed with decreasing particle size, one-way ANOVA indicated that the differences among fractions were not statistically significant (F = 3.841, *p* = 0.084). Tukey’s post-hoc comparisons also showed no significant differences between split and crushed (*p* = 0.273), split and fine (*p* = 0.075), or crushed and fine (*p* = 0.592).

### 3.3. Physicochemical Properties of Unfermented and Fermented Lupin Flour

Following fermentation, the fermented lupin products were freeze-dried and subsequently milled and sieved to obtain a consistent powder size prior to further physicochemical and functional analyses. Thus, the particle-size treatment in this study refers to the initial substrate particle size used during fermentation, rather than the particle size of the final flour used for subsequent analyses. To confirm the particle-size distribution of the fermented products after standardised post-fermentation processing, the samples were analysed using a Mastersizer. The D10 values ranged from 12.83 to 13.70 µm, D50 values from 166.67 to 171.67 µm, and D90 values from 710.00 to 799.67 µm. No significant differences were observed among the unfermented, fermented split, fermented crushed, and fermented fine samples for D10, D50, or D90 (*p* > 0.05). These results confirm that the final powders used for subsequent analyses had comparable particle-size distributions, irrespective of their initial particle-size.

#### 3.3.1. Proximate Composition of Fermented Lupin Flours

The effect of fermentation on lupin’s proximate contents is shown in Table 2.

Fermentation significantly affected crude protein, fat, insoluble dietary fibre (IDF), soluble dietary fibre (SDF), starch, and calculated carbohydrate contents (*p* < 0.05), whereas moisture and ash contents remained statistically unchanged among treatments. Moisture content was relatively stable across all samples, indicating that the freeze-drying process produced flours with comparable residual moisture levels irrespective of particle size. Similarly, ash content showed only minor variation following fermentation, with no significant differences observed among the flour fractions. Crude protein content varied among the different particle-size treatments following fermentation. Among the fermented samples, the fine fraction exhibited the highest protein content (43.5%), followed by the crushed fraction (39.5%), whereas the split fraction showed the lowest value (32.2%). The unfermented control had an intermediate protein content of 37.1%. Thus, fermentation did not result in a uniform increase in protein content across all particle-size treatments, but the fermented fine and crushed fractions showed higher protein concentrations than the unfermented control. A reduction in crude fat content was observed following fermentation across all particle-size fractions. The decrease was more pronounced in the crushed and fine fractions than in the split fraction, with the fermented fine flour exhibiting the lowest fat content (2.0%). Fermentation also altered the distribution of dietary fibre fractions. Insoluble dietary fibre decreased substantially in all fermented samples compared with the unfermented flour, with the split fraction retaining the greatest proportion of IDF among the fermented flours. Conversely, soluble dietary fibre varied considerably among particle sizes, reaching its highest level in the crushed fraction, while the split fraction showed the lowest SDF content. Starch content remained consistently low across all samples, although a slight reduction was observed following fermentation, particularly in the fine fraction. Calculated carbohydrate content also differed significantly among treatments, with the fermented split flour exhibiting the highest carbohydrate content and the fermented fine flour the lowest. Overall, particle size influenced the compositional characteristics of fermented lupin flour. The fine fraction was characterised by higher protein and lower fat contents, the crushed fraction contained the greatest proportion of soluble dietary fibre, and the split fraction retained comparatively higher insoluble dietary fibre and carbohydrate contents.

#### 3.3.2. Amino Acid Profiling

The amino acid composition of unfermented and fermented lupin flour fractions is presented in Table 3. Fermentation and particle size significantly influenced the concentrations of several amino acids, with different responses observed among essential and non-essential amino acids.

The total essential amino acid (EAA) content ranged from 11.15 ± 0.57 mg/100 mg in fermented split flour to 12.33 ± 0.96 mg/100 mg in fermented crushed flour. Fermented crushed and fine fractions showed higher total EAA contents compared with fermented split flour. Among the essential amino acids, phenylalanine, isoleucine, leucine, lysine, methionine, threonine and valine showed significant differences among treatments. Phenylalanine content ranged from 1.57 ± 0.02 mg/100 mg in fermented split flour to 1.78 ± 0.01 mg/100 mg in fermented fine flour. Methionine showed an increasing trend after fermentation, with the highest concentrations observed in fermented crushed (0.42 ± 0.02 mg/100 mg) and fermented fine (0.45 ± 0.02 mg/100 mg) fractions. Lysine content was highest in unfermented flour (1.95 ± 0.02 mg/100 mg), while fermented split flour showed the lowest value (1.74 ± 0.02 mg/100 mg).

The total non-essential amino acid (NEAA) content ranged from 22.93 ± 0.83 mg/100 mg in fermented crushed flour to 28.29 ± 0.63 mg/100 mg in unfermented flour. Fermented fine flour showed a higher NEAA content (25.15 ± 0.68 mg/100 mg) compared with fermented split and crushed fractions. Aspartic acid, serine, glutamic acid, proline, glycine, alanine, tyrosine, arginine and cysteine showed differences among treatments. Glutamic acid was the most abundant non-essential amino acid in all samples, with concentrations ranging from 7.36 ± 0.02 mg/100 mg in fermented crushed flour to 10.72 ± 0.03 mg/100 mg in unfermented flour. Arginine concentration decreased following fermentation, with values ranging from 4.45 ± 0.10 mg/100 mg in unfermented flour to 1.84 ± 0.11 mg/100 mg in fermented fine flour.

The fermented crushed and fine fractions showed higher glycine and alanine concentrations compared with unfermented flour. Glycine increased from 1.60 ± 0.04 mg/100 mg in unfermented flour to 1.75 ± 0.12 mg/100 mg and 2.08 ± 0.03 mg/100 mg in fermented crushed and fine fractions, respectively. Alanine increased from 1.36 ± 0.12 mg/100 mg in unfermented flour to 1.87 ± 0.04 mg/100 mg and 1.99 ± 0.02 mg/100 mg in fermented crushed and fine fractions, respectively.

#### 3.3.3. Colour

Colour of lupin was significantly affected by fermentation, and the colour measurements is presented in Table 4. Fermentation and particle size resulted in significant differences in lightness (L*) and redness (a*) values, whereas yellowness (b*) values remained relatively unchanged among treatments.

Fermentation and particle size had a significant effect on lightness (L*) (*p* < 0.05), whereas yellowness (*b) and and redness (a*) remained unaffected. Lightness differed among the flour fractions, with the fermented crushed fraction exhibiting the highest *L* value. In contrast, the unfermented and fermented fine flours showed similar lightness values, while the fermented split fraction displayed an intermediate value. Overall, fermentation produced only slight changes in sample lightness. Redness (a*) is also non-significantly affected among treatments. b* values remained relatively constant across all samples, with no significant differences observed among the unfermented and fermented flour fractions. The total colour difference (ΔE) further indicated that the fermented crushed fraction exhibited the greatest overall colour difference relative to the unfermented flour (2.75 ± 0.72), followed by the fermented split fraction (1.38 ± 0.57), whereas the fermented fine fraction showed the smallest colour difference (0.63 ± 0.43). These results suggest that the greater overall colour change observed in the crushed fraction was mainly associated with changes in lightness and redness, while the relatively small ΔE of the fine fraction reflected its colour profile being closer to that of the unfermented flour.

### 3.4. Functional Properties

Functional properties of fermented lupin flours are presented in Table 5 and Table 6**,** and the explanation of each functional property is described in the following subsections.

#### 3.4.1. Water and Oil Holding Capacities

The WHC values varied among the samples, with fermented crushed flour exhibiting the highest water retention capacity compared with the other fractions. The unfermented, fermented split, and fermented fine fractions showed similar WHC values, with no significant differences observed among these samples. Overall, fermentation resulted in a slight improvement in WHC, particularly in the crushed fraction. Oil holding capacity showed a different trend compared with WHC. The unfermented flour exhibited the highest OHC, while fermented split flour showed the lowest oil retention capacity. The fermented crushed and fine fractions displayed intermediate OHC values and were not significantly different from either the unfermented or split fractions.

#### 3.4.2. Least Gelation Concentration

The least gelation concentration (LGC) was reduced following fermentation, indicating improved gel-forming ability of the fermented flours compared with the unfermented sample. The fermented split fraction showed the lowest LGC value, whereas the unfermented flour required the highest concentration for gel formation. The crushed and fine fractions exhibited intermediate LGC values and were not significantly different from either the split or unfermented samples.

#### 3.4.3. Functional Properties Across Different pH

Emulsifying activity (EA) varied significantly among the flour fractions across all pH conditions. The fermented crushed fraction exhibited the highest emulsifying activity at all tested pH values, followed by the fermented split fraction. The unfermented and fermented fine fractions showed comparatively lower emulsifying activity, with the fine fraction displaying the lowest values at acidic pH conditions. Across all samples, emulsifying activity was lowest at pH 4 and increased under neutral to alkaline conditions. Emulsifying stability (ES) showed similar pH-dependent variation among treatments. The fermented split fraction demonstrated the highest emulsifying stability across all pH values, whereas the fermented crushed fraction exhibited the lowest stability. The fermented fine fraction showed intermediate stability, while the unfermented flour generally displayed lower stability compared with fermented split and fine fractions. Emulsifying stability increased as pH increased from acidic to alkaline conditions for most samples.

Foaming capacity (FC) differed significantly among the samples, with the fermented crushed fraction showing the highest foaming capacity across all pH conditions. The fermented split fraction exhibited the second-highest values, while the fermented fine fraction showed the lowest foaming capacity at pH 2 and 4. Similar to emulsifying activity, foaming capacity increased with increasing pH, with the highest values observed at pH 8. Foaming stability (FS) followed a similar trend to foaming capacity, with significant differences observed among treatments. The fermented split fraction exhibited the highest foaming stability across all pH conditions, whereas the fermented crushed fraction showed the lowest stability. The fermented fine and unfermented samples showed intermediate stability values. Foaming stability increased under higher pH conditions for all samples.

Protein solubility was strongly influenced by pH and fermentation treatment. All samples showed the lowest protein solubility near pH 4, while the highest solubility was observed at pH 6 and 8. Among the fermented fractions, the crushed sample exhibited the highest protein solubility across the tested pH range, followed by the fermented split fraction. The fermented fine fraction showed lower solubility compared with the other fermented samples, particularly at neutral and alkaline pH conditions.

### 3.5. Rheological Properties

The rheological behaviour of unfermented and fermented lupin flour pastes was evaluated using temperature and frequency sweep measurements. A strain value of 0.03% was selected for subsequent rheological analysis based on measurements conducted within the linear viscoelastic region (LVR) of the samples (Supplementary Materials).

#### 3.5.1. Temperature Sweep Analysis

**Figure 2:**
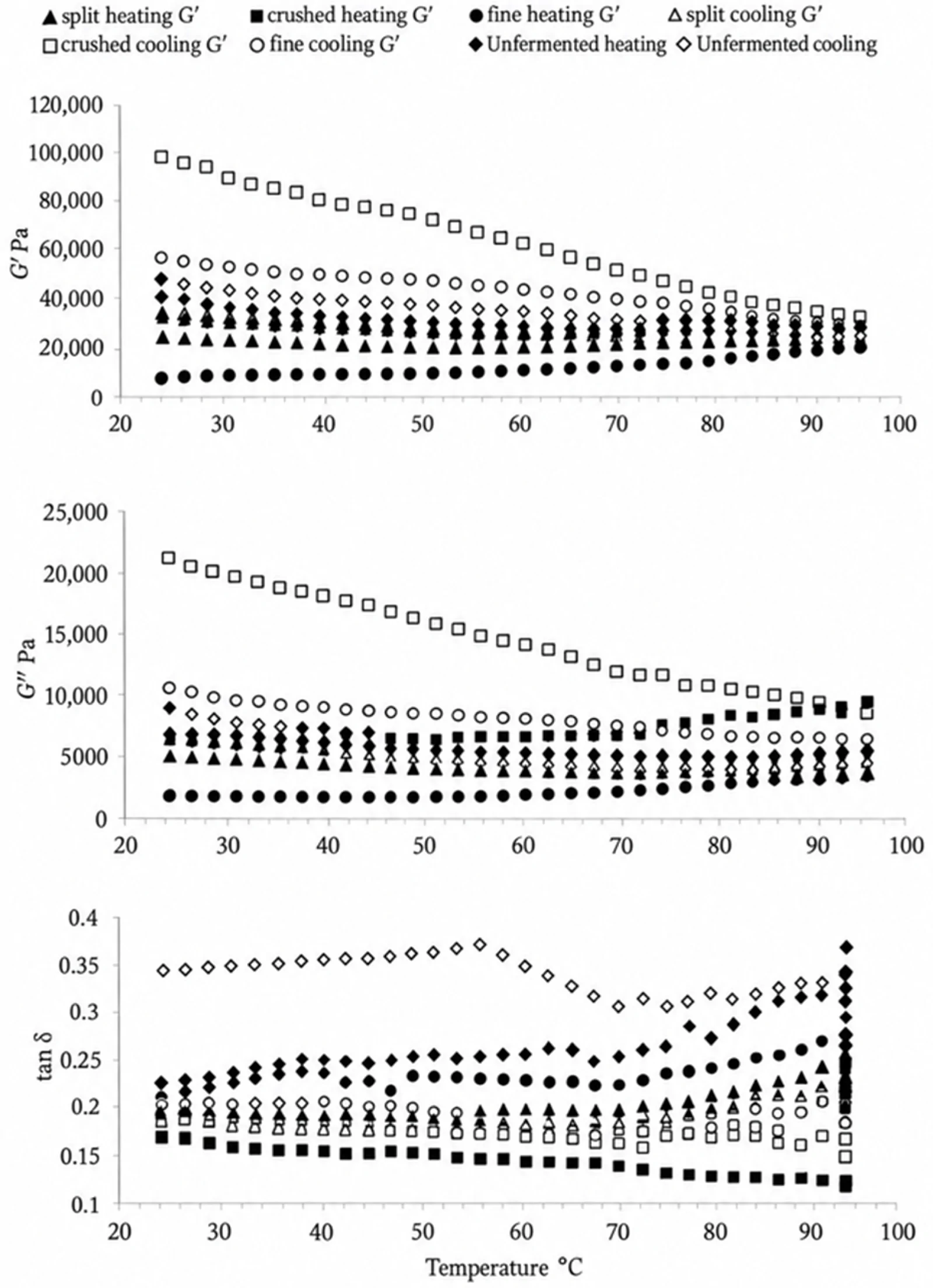
Temperature sweeps of lupin flour pastes.

The temperature-dependent rheological behaviour of lupin pastes is shown in Figure 2. Temperature sweep analysis was performed to evaluate the viscoelastic behaviour and thermal stability of 25% (*w*/*v*) unfermented and fermented lupin flour dispersions with different particle sizes during heating (25–95 °C) and subsequent cooling (Figure 2). Throughout the temperature sweep, all samples exhibited storage modulus (G′) values consistently greater than loss modulus (G″), while tan δ remained below 1, indicating that elastic behaviour predominated over viscous behaviour regardless of particle size or fermentation treatment.

Among the samples, the fermented crushed flour exhibited the highest G′ values throughout both heating and cooling, followed by the unfermented flour, whereas the fermented split and fine flours displayed comparatively lower G′ values. A gradual decrease in G′ was observed with increasing temperature for all samples, followed by partial recovery during cooling. Similar trends were observed for G″, with the crushed flour maintaining the highest values across the temperature range. The tan δ values differed among the samples. The fermented crushed flour consistently exhibited the lowest tan δ values (approximately 0.12–0.17), whereas the unfermented flour showed the highest tan δ values (approximately 0.29–0.36). The fermented split and fine flours exhibited intermediate tan δ values throughout the temperature sweep.

#### 3.5.2. Frequency Sweep Analysis

**Figure 3:**
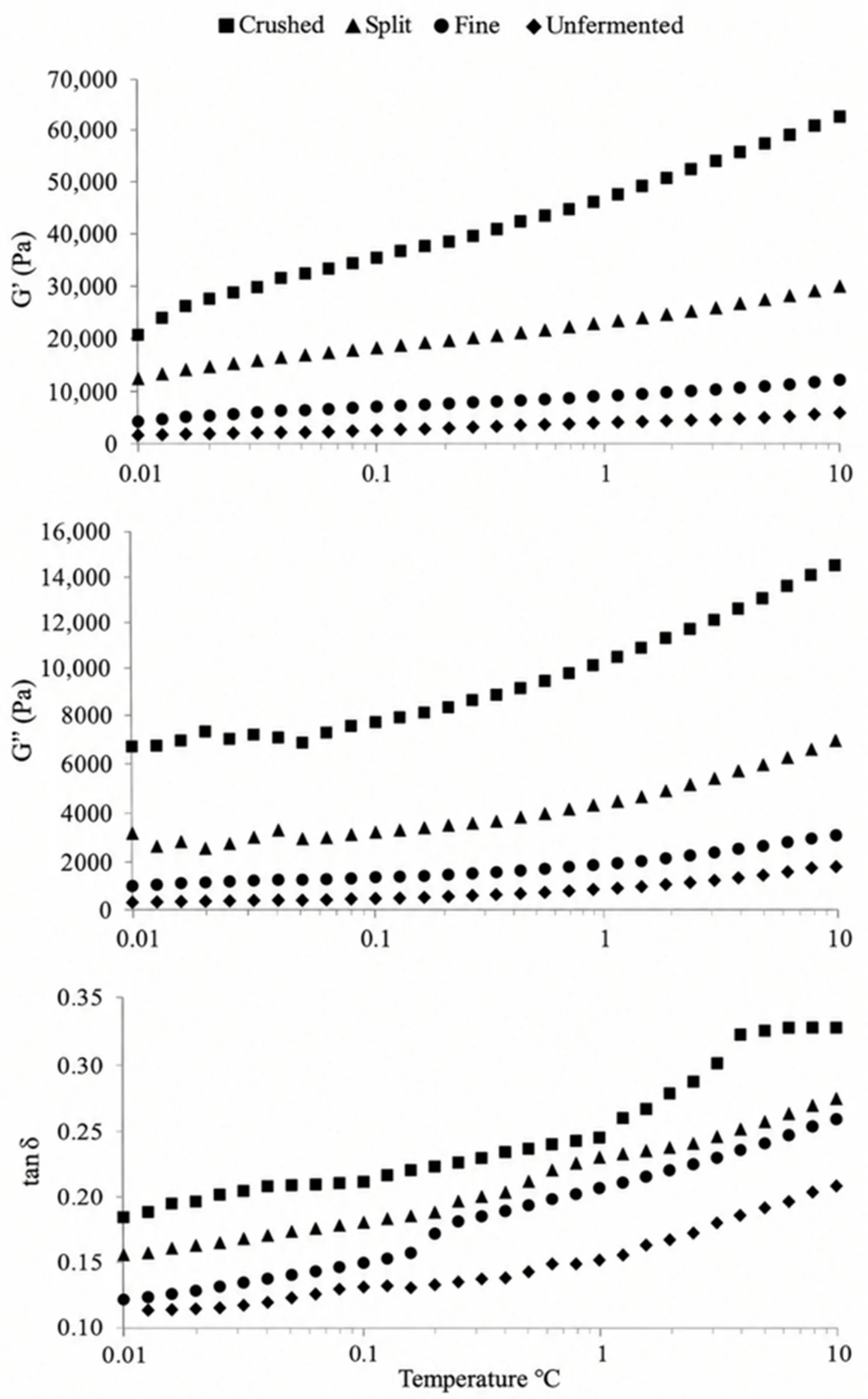
Frequency sweeps of lupin flour pastes.

The frequency-dependent rheological behaviour of lupin pastes is presented in Figure 3.

All samples exhibited frequency-dependent behaviour, with both G′ and G″ increasing as frequency increased from 0.01 to 10 Hz. Across the tested frequency range, G′ values remained higher than G″ values for all samples, confirming the elastic-dominant nature of the lupin paste systems. The fermented crushed fraction showed the highest G′ and G″ values across the frequency range, followed by fermented split, fermented fine, and unfermented samples. The differences among samples were maintained throughout the frequency sweep, with fermented crushed flour demonstrating the greatest viscoelastic strength.

### 3.6. Fourier Transform Infrared Spectroscopy

**Figure 4:**
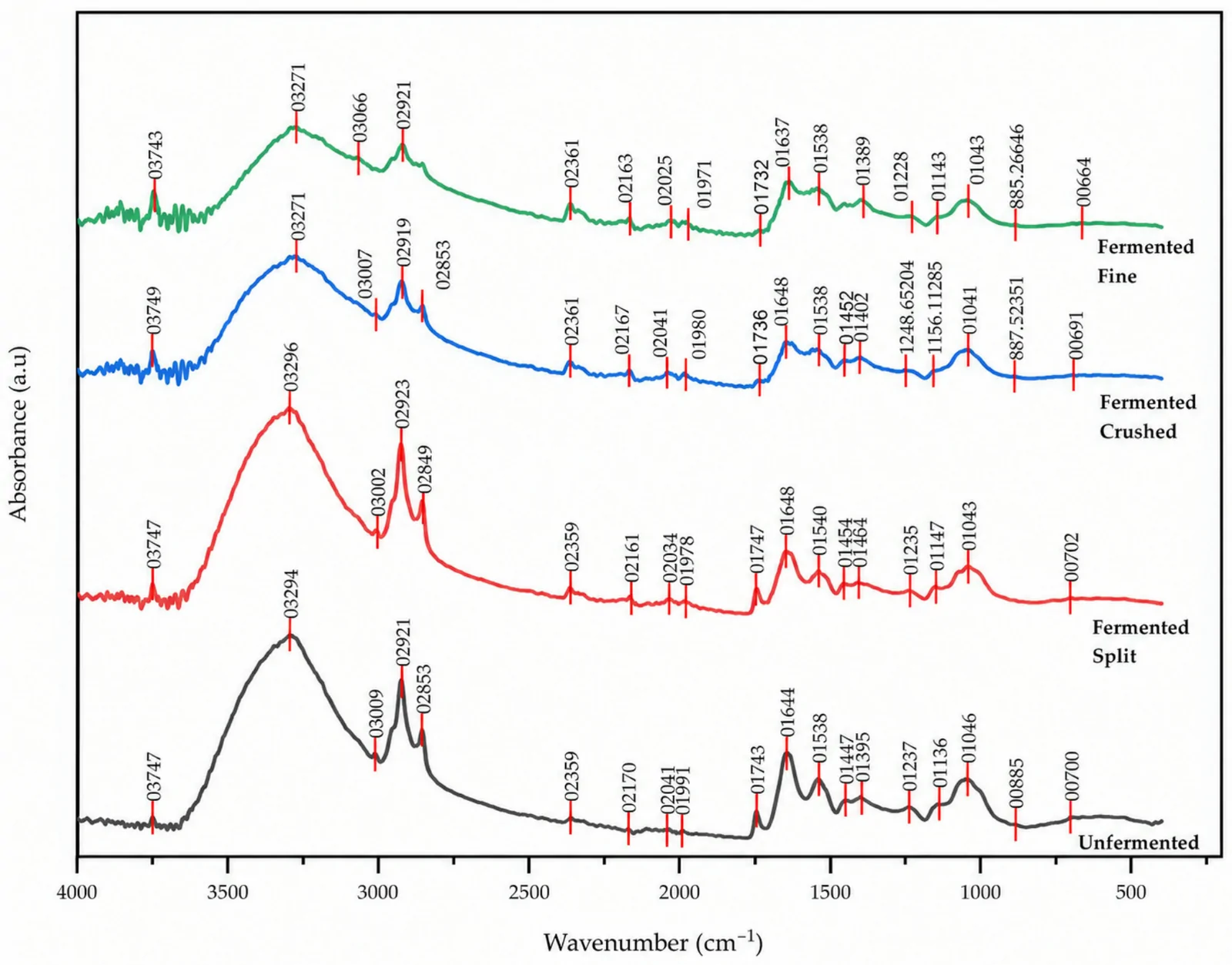
Changes in FTIR spectra of unfermented vs. fermented lupin counterparts.

FTIR spectroscopy was used to evaluate molecular changes in unfermented and fermented lupin flour fractions. The FTIR spectra of all samples are presented in Figure 4. All samples showed similar overall spectral profiles, with characteristic absorption bands associated with hydroxyl, lipid, protein, and carbohydrate components.

The main absorption regions observed included the broad O–H/N–H stretching region (approximately 3600–3000 cm^−1^), lipid-associated C–H stretching region (approximately 2925 and 2850 cm^−1^), carbonyl stretching region (approximately 1740 cm^−1^), Amide I region (1700–1600 cm^−1^), Amide II region, and carbohydrate fingerprint region. Following fermentation, changes were observed in peak position and intensity among different particle size fractions. The broad O–H/N–H stretching band shifted from approximately 3303 cm^−1^ in unfermented flour to approximately 3275–3289 cm^−1^ in fermented samples. Changes were also observed in lipid-associated regions. The absorption bands corresponding to CH_2_ asymmetric and symmetric stretching vibrations near 2925 and 2850 cm^−1^ showed differences among treatments following fermentation. The carbonyl region around 1740 cm^−1^ showed changes in intensity among samples, with greater changes observed in fermented crushed and fine fractions compared with unfermented flour. The Amide I region showed differences in peak position among samples. The unfermented sample exhibited an Amide I peak around 1641 cm^−1^, while fermented crushed and fine fractions showed shifts towards approximately 1646 cm^−1^. Changes were also observed in the Amide II region following fermentation.

#### 3.6.1. Protein Secondary Structure Analysis by Amide I Deconvolution

The Amide I region (1700–1600 cm^−1^) was further analysed using Gaussian deconvolution to estimate the relative proportions of protein secondary structures (Table 7). β-sheet structures remained the predominant secondary structure component in all samples, accounting for approximately 34–36% of total Amide I area. The unfermented sample showed the highest β-sheet proportion (36.25%), while fermented split, crushed, and fine fractions showed values of 34.35%, 35.92%, and 35.13%, respectively. The α-helix content increased in fermented crushed and fine fractions compared with unfermented flour. The fermented crushed fraction showed the highest α-helix proportion (19.78%), followed by fermented fine (19.44%), unfermented (16.11%), and fermented split (15.34%) samples. Random coil structures ranged from 13.48% to 15.25%. The unfermented sample exhibited the highest random coil content (15.25%), whereas fermented crushed and fine fractions showed lower values (13.48% and 13.52%, respectively). β-turn structures accounted for approximately 27–31% of the total secondary structure. The highest β-turn proportion was observed in fermented split flour (30.74%), followed by unfermented flour (29.26%), fermented crushed (27.77%), and fermented fine fractions (27.35%). Aggregated β-sheet structures represented the smallest proportion of secondary structures, ranging from 2.86% to 5.76%. The fermented split fraction showed the highest aggregated strand content (5.76%), while the unfermented sample showed the lowest value (2.86%).

#### 3.6.2. Changes in Functional Group Regions

The fingerprint region showed differences among treatments following fermentation. Changes were observed in bands associated with carbohydrate and polysaccharide components, particularly among fermented crushed and fine fractions. Overall, FTIR analysis demonstrated that fermentation and particle size affected the spectral characteristics of lupin flour, with differences observed in regions associated with proteins, lipids, and carbohydrates.

### 3.7. Differential Scanning Calorimetry

Differential scanning calorimetry (DSC) was used to evaluate the thermal transition behaviour of unfermented and fermented lupin flour fractions. The DSC thermograms showed two endothermic peaks for all samples (Table 8), corresponding to the thermal transitions of lupin protein fractions.

The first endothermic peak occurred between 88.40 ± 10.02 °C and 89.51 ± 80.43 °C across all samples. No significant differences were observed in the denaturation temperature (Td) of Peak 1 among treatments (*p* > 0.05). The fermented crushed fraction exhibited the highest Peak 1 temperature (89.51 ± 8.43 °C), while the unfermented sample showed the lowest value (88.40 ± 10.02 °C). The second endothermic peak occurred between 102.41 ± 10.24 °C and 105.47 ± 7.20 °C. Similar to Peak 1, no significant differences were observed in the denaturation temperature of Peak 2 among samples. The fermented fine fraction showed the highest Peak 2 temperature (105.47 ± 7.20 °C), whereas the unfermented sample showed the lowest value (102.41 ± 10.24 °C). Although denaturation temperatures remained relatively consistent, significant differences were observed in enthalpy change (ΔH) values among treatments. For Peak 1, the highest ΔH was observed in fermented crushed flour (6.72 ± 2.11 J/g), followed by fermented split flour (5.10 ± 1.72 J/g), unfermented flour (3.30 ± 1.44 J/g), and fermented fine flour (2.32 ± 1.08 J/g). A similar pattern was observed for Peak 2. Fermented crushed flour exhibited the highest ΔH value (4.71 ± 0.01 J/g), followed by fermented split flour (3.11 ± 1.12 J/g), unfermented flour (2.62 ± 1.05 J/g), and fermented fine flour (1.77 ± 0.52 J/g). Overall, fermentation and particle size significantly influenced the enthalpy changes associated with protein thermal transitions, while the denaturation temperatures of the major thermal transitions remained relatively unchanged.

### 3.8. Multivariate Analysis: Principal Component Analysis and Pearson Correlation

**Figure 5:**
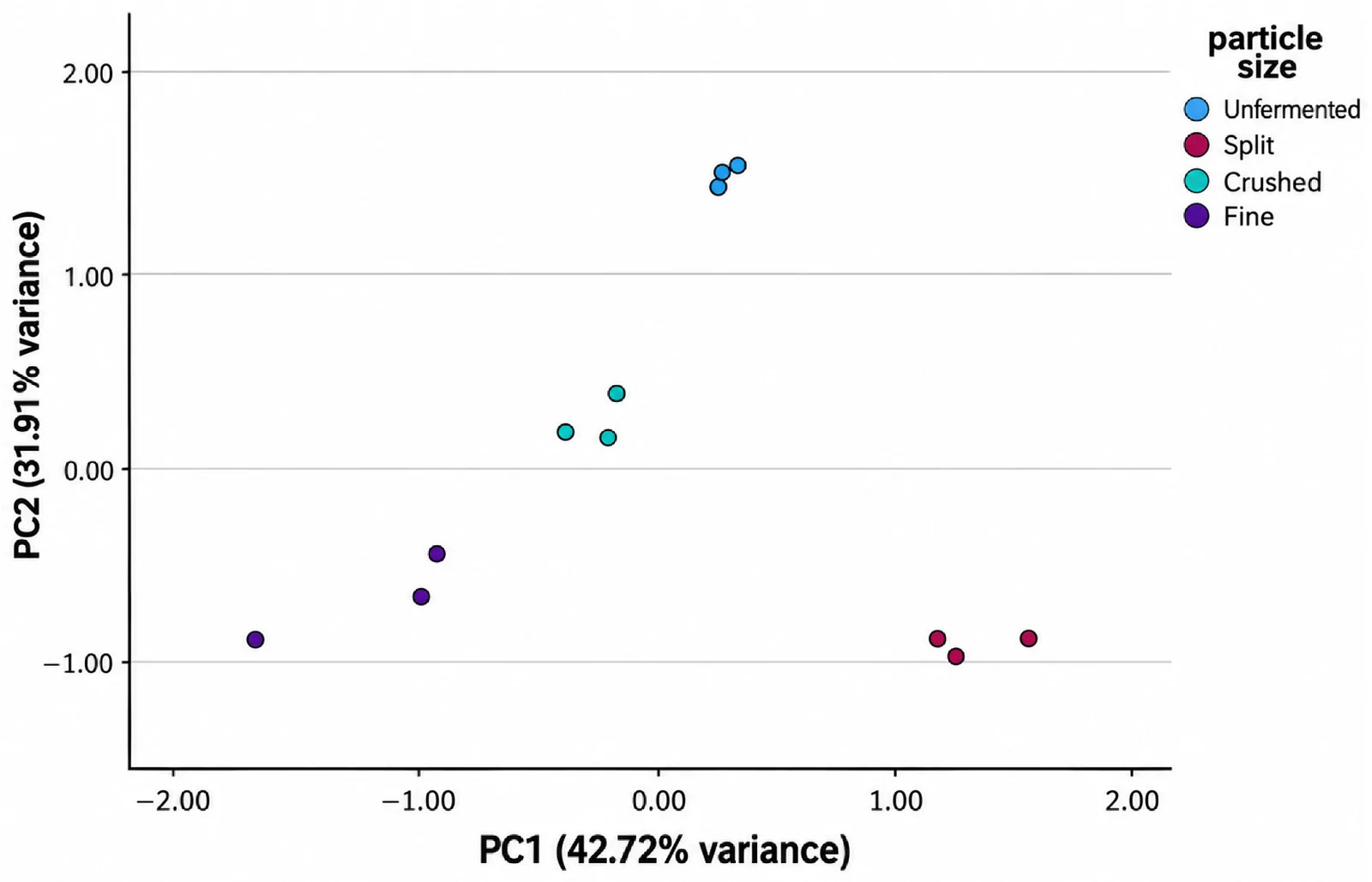
Score plot of (PC1 vs. PC2) of lupin samples with different particle sizes and fermentation treatments.

Principal component analysis (PCA) was performed to evaluate the relationships among physicochemical, structural, and functional properties of unfermented and fermented lupin fractions. The first two principal components explained 74.6% of the total variance, with PC1 and PC2 accounting for 42.7% and 31.9%, respectively (Figure 5).

**Figure 6:**
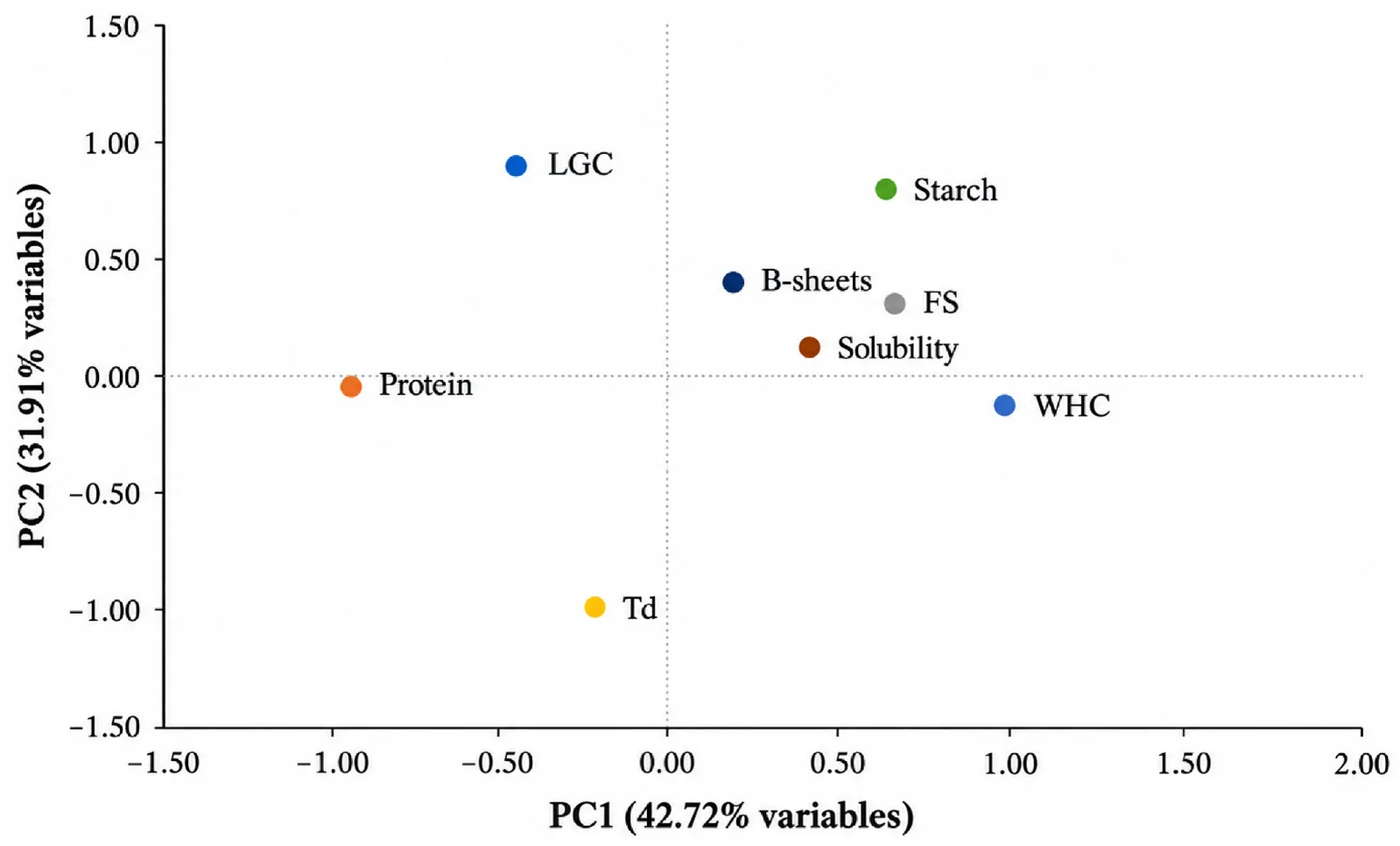
Loading plot of (PC1 vs. PC2) of lupin samples with different particle sizes and fermentation treatments.

The PCA score plot showed separation among samples according to fermentation treatment and particle size. The fermented crushed sample was positioned separately from the other treatments, whereas the split and fine fractions showed different distribution patterns. The loading (Figure 6) plot indicated that functional properties, including protein solubility, water holding capacity, and foaming properties, contributed strongly to sample differentiation.

Pearson correlation analysis demonstrated significant relationships among several measured parameters (Supplementary Materials). Protein solubility showed strong positive correlations with emulsifying activity and foaming capacity (r > 0.94, *p* < 0.01). Correlations were also observed between structural parameters and functional properties, indicating associations among molecular characteristics and measured functionality.

## 4. Discussion

### 4.1. Particle Size as a Critical Factor Controlling R. oligosporus Fermentation

Particle size is an important processing parameter that determines substrate accessibility, microbial colonisation, and the extent of biochemical transformation during fermentation [38]. In this study, milling created three distinct lupin fractions, allowing evaluation of how physical structure influences fungal fermentation performance. The results demonstrated that particle size reduction did not simply enhance fermentation efficiency in a linear manner. Instead, an intermediate particle size (crushed fraction) provided the most favourable conditions for fungal modification while maintaining functional characteristics.

This was also reflected in the dry matter (DM) characteristics during fermentation. Although fermentation time did not significantly affect DM content (*p* = 0.137), particle-size fraction had a significant effect (*p* = 0.008), with the split fraction exhibiting the highest mean DM content (24.25 ± 0.15%) and the fine fraction the lowest (22.86 ± 0.20%). The split fraction had significantly higher DM content than the fine fraction, whereas the crushed fraction was intermediate and did not differ significantly from either treatment. These differences indicate that the initial particle structure influenced the dry matter characteristics of the fermentation matrix, with progressively finer particles associated with lower DM content. Despite these differences in DM content, overall DM recovery remained high across all fractions, ranging from 94.53 ± 1.37% in the fine fraction to 97.20 ± 0.84% in the split fraction, and the effect of particle size on DM recovery was not statistically significant (*p* = 0.084). Thus, the lower DM content observed for the finer fraction was not accompanied by a statistically significant increase in overall dry matter loss. This distinction is important because it suggests that the differences among fractions were more likely related to differences in moisture distribution and the physical characteristics of the fermentation matrix than to substantial differences in substrate disappearance. The slightly greater numerical DM loss in the fine fraction may nevertheless indicate a tendency towards greater dry matter utilisation or material loss during fermentation, consistent with its greater degree of compositional modification observed elsewhere in this study.

Reduction of particle size generally increases surface area and decreases diffusion distance, which can improve moisture absorption, enzyme accessibility, and fungal penetration into plant tissues [39]. This explains why the crushed and fine fractions showed greater compositional modification compared with the larger split fraction. Increased accessibility likely promoted fungal secretion of hydrolytic enzymes, including proteases, cellulases, and lipases, resulting in greater modification of lupin macromolecules [40]. However, further reduction to the fine fraction did not result in continuous improvement. Excessively small particles may promote particle aggregation and reduce pore spaces within the fermentation matrix, limiting oxygen transfer and fungal growth [41]. Although *R. oligosporus* can tolerate variations in oxygen availability, oxygen limitation can influence fungal metabolism and enzyme production [42]. Therefore, successful fermentation requires a balance between increasing substrate accessibility and maintaining a suitable physical structure for fungal development [42].

### 4.2. Effect of Fermentation and Particle Size on Compositional and Nutritional Changes

Fermentation with *R. oligosporus* resulted in changes in the composition of lupin flour, with the magnitude and direction of these changes varying according to the initial substrate particle size. The fermented fine and crushed fractions exhibited higher apparent protein contents than the unfermented control, whereas the fermented split fraction showed a lower protein content. Therefore, fermentation did not result in a uniform increase in protein concentration across all treatments. The higher apparent protein concentrations observed in the crushed and fine fractions may be partly associated with changes in the relative proportions of non-protein components during fermentation rather than direct protein synthesis [43]. This distinction is important because fungal growth can increase the relative proportion of protein through utilisation or transformation of other substrate components [43]. However, as protein mass balance and fungal biomass formation were not directly measured in the present study, these mechanisms should be considered as possible explanations rather than direct evidence of protein synthesis or degradation.

A reduction in lipid content was observed following fermentation across all particle-size fractions, with greater reductions in the crushed and fine fractions. *R. oligosporus* is known to produce lipolytic enzymes that can contribute to lipid hydrolysis during fermentation [44]. The greater reduction observed in the smaller particle fractions may be associated with increased substrate accessibility resulting from greater exposed surface area [44]. Nevertheless, lipase activity and direct lipid utilisation were not measured in the present study; therefore, the observed reduction in lipid content is interpreted as an outcome of fermentation rather than direct evidence of enzymatic lipid degradation.

Dietary fibre composition also varied following fermentation. In particular, the crushed fraction showed a marked increase in soluble dietary fibre accompanied by a reduction in insoluble dietary fibre. This pattern may indicate modification or solubilisation of structural polysaccharides during fermentation. Fungal carbohydrate-degrading enzymes, including cellulolytic enzymes, may contribute to the modification of plant cell-wall components [45]. However, because enzyme activities and direct conversion pathways were not measured, the observed changes in IDF and SDF should be interpreted as compositional changes associated with fermentation rather than direct evidence of enzymatic conversion. The increase in soluble fibre may nevertheless be relevant to functional properties such as hydration and viscosity development [46,47]. Changes in amino acid composition further indicated that fermentation altered the amino acid profile of lupin flour. The reduction in arginine across the fermented samples may reflect its utilisation during fungal growth, consistent with previous reports of arginine metabolism by filamentous fungi [48,49]. In contrast, increases in selected amino acids, including glycine, alanine and methionine, particularly in the smaller particle fractions, may be associated with differences in protein hydrolysis and amino acid metabolism during fermentation. However, because enzyme activity and substrate utilisation were not directly monitored, these proposed mechanisms cannot be confirmed from the present data alone. Overall, the results demonstrate that fermentation altered the compositional profile of lupin flour and that the magnitude of these changes depended on the initial substrate particle size. These findings support the role of substrate structure in influencing the outcome of fermentation, while direct measurements of fungal biomass, substrate utilisation and enzyme activity in future studies would provide further insight into the mechanisms responsible for these changes.

### 4.3. Molecular and Structural Modification of Lupin Proteins and Matrix Components

The FTIR and DSC results indicate that fermentation induced controlled molecular restructuring rather than extensive destruction of lupin macromolecules. This is an important observation because maintaining structural integrity is essential when developing functional plant-based ingredients [50]. Changes in the Amide I region indicated modification of protein conformation following fungal fermentation. The reduction in β-sheet structures and changes in α-helix and β-turn proportions suggest partial unfolding and rearrangement of lupin storage proteins [51]. Lupin proteins, particularly conglutins, possess highly organised globular structures stabilised by hydrogen bonding and hydrophobic interactions [52]. Partial hydrolysis by fungal proteases may expose previously inaccessible regions while allowing subsequent molecular rearrangement [53].

Importantly, the extent of modification depended on particle size. The crushed fraction showed structural changes associated with improved functionality while maintaining a high proportion of ordered structures. In contrast, the fine fraction exhibited evidence of greater modification but did not translate into improved functional performance. This indicates that excessive molecular disruption may reduce the ability of proteins to participate in intermolecular interactions required for emulsification, foaming, and gel formation [54]. The DSC results support this interpretation. Similar denaturation temperatures among treatments indicate that fermentation did not substantially alter the fundamental thermal stability of lupin proteins. However, differences in enthalpy suggest variation in structural organisation [55]. The higher enthalpy observed in the fermented crushed fraction indicates that greater energy was required for thermal unfolding, suggesting stronger or more extensive intermolecular interactions within the protein network [56]. Together, FTIR and DSC demonstrate that optimal fermentation involves controlled protein modification rather than maximum degradation. Maintaining an appropriate balance between accessibility and structural preservation is therefore essential for developing functional fermented lupin ingredients.

### 4.4. Relationship Between Structural Modification and Techno-Functional Properties

The functional performance of fermented lupin flour appeared to depend on the extent of modification occurring during fermentation and the structural characteristics associated with the initial substrate particle size. The particle-size results showed that fermentation increased the apparent Feret diameter of the lupin fractions, with significant increases observed for the split and crushed fractions, while the change in the fine fraction was not significant. These changes were accompanied by shifts in particle-size distribution towards larger size classes, particularly for the split and crushed fractions. Importantly, following freeze-drying, milling and sieving, no significant differences in particle-size distribution were observed among the final powders. Therefore, the functional differences observed among the fermented fractions cannot be attributed simply to differences in the particle size of the final flour. Rather, they are more likely related to differences in the structural and compositional changes that occurred during fermentation as a consequence of the initial particle-size treatment.

Water holding capacity (WHC) increased following fermentation, with the crushed fraction showing the highest numerical value. This enhancement may be associated with changes in protein and fibre characteristics during fermentation. Partial protein hydrolysis may increase the accessibility of polar groups, while modification of dietary fibre may alter the availability of hydrophilic sites for water interaction [56,57,58]. Similar improvements in hydration properties have been reported for fermented legume ingredients, where microbial enzymes modify protein and cell-wall polysaccharides and increase water accessibility [59]. However, the relationship between initial particle size and WHC was not linear. Although the fine fraction had the highest apparent protein content, its WHC was lower than that of the crushed fraction. This suggests that the functional response depended on the balance between molecular modification and the structural organisation of the fermented material rather than on protein content alone. Thus, the crushed fraction appeared to provide a favourable combination of molecular accessibility and structural characteristics for water retention.

Protein solubility provided further evidence of differences in the extent of functional modification among the fractions. All samples exhibited the expected pH-dependent solubility behaviour, with lower solubility around the isoelectric region and greater solubility under neutral and alkaline conditions [60]. Fermentation increased protein solubility, with the crushed fraction showing the most favourable numerical response. This may be related to partial protein hydrolysis and changes in protein conformation that increase the accessibility of charged groups [61]. Greater protein solubility and dispersion can facilitate protein migration and rearrangement at oil–water and air–water interfaces, potentially contributing to improved emulsifying and foaming properties [62]. The favourable emulsifying activity and foaming capacity of the crushed fraction therefore suggest that its initial particle size may have promoted a suitable degree of fermentation-induced protein modification while retaining sufficient structural organisation. In contrast, emulsifying and foaming stability did not necessarily follow the same pattern as activity. The split fraction showed greater stability despite lower activity than the crushed fraction, indicating that stability may depend on additional structural characteristics of the fermented material, including the contribution of fibre and residual particle structure [63].

The rheological results further demonstrated that fermentation altered the functional behaviour of the lupin flours. All dispersions exhibited predominantly elastic behaviour during heating and cooling, with G′ remaining higher than G″ and tan δ values below 1, indicating that the elastic component dominated the viscoelastic response [64]. The fermented crushed flour exhibited the highest G′ together with relatively low tan δ values, indicating a comparatively strong and elastic gel network. This behaviour is consistent with the favourable hydration and protein functionality observed for the crushed fraction, suggesting that the extent of fermentation-induced modification and the resulting structural characteristics supported formation of a more cohesive network. In contrast, fermented fine and split flours exhibited lower G′ values than the unfermented flour, indicating that fermentation did not enhance gel strength uniformly across the initial particle-size treatments. Although the crushed fraction showed the most favourable numerical rheological performance, the differences should be interpreted together with the statistical results for the other functional properties rather than as evidence of a universally superior fraction.

Interestingly, the unfermented flour exhibited higher G′ values than the fermented fine and split fractions but also showed higher tan δ values throughout the temperature sweep. This indicates that the native flour formed a relatively stiff network while exhibiting a greater contribution from viscous dissipation compared with the fermented samples. Therefore, G′ alone does not fully describe network quality, and consideration of both G′ and tan δ provides a more complete description of the viscoelastic behaviour [65]. The decrease in G′ and G″ during heating indicates weakening of the existing intermolecular network as increasing temperature enhanced molecular mobility and disrupted non-covalent interactions [66]. During cooling, partial recovery of both moduli suggests re-establishment of intermolecular interactions and partial reconstruction of the network.

Overall, the results demonstrate that the initial particle size used during fermentation influenced the subsequent physicochemical and techno-functional characteristics of lupin flour, even though the final powders were standardised to comparable particle-size distributions before analysis. The crushed fraction generally showed the most favourable numerical performance across several properties, particularly WHC, emulsifying and foaming characteristics, and rheological behaviour. However, the absence of a consistent advantage across all functional properties indicates that no single initial particle size can be considered universally optimal. Instead, the suitability of fermented lupin flour is likely to depend on the specific functional requirements of the intended food application. Further studies should therefore evaluate the performance of these fermented flours during food processing and storage and assess their sensory properties to establish application-specific suitability.

### 4.5. Integrated Interpretation Using PCA and Pearson Correlation Analysis

Multivariate analysis provided an integrated assessment of the relationships among fermentation treatment, particle size, and the measured physicochemical, structural, and functional properties of the lupin samples. PCA was performed using the standardised selected variables, including protein content, starch content, low-gelation concentration (LGC), water-holding capacity (WHC), protein solubility, foaming stability (FS), β-sheet content, and denaturation temperature (Td). PC1 and PC2 explained 42.7% and 31.9% of the total variance, respectively, accounting for 74.6% of the overall variation. The loading plot showed that WHC, starch, FS, and solubility were associated with the positive direction of PC1, whereas protein content and LGC were associated with its negative direction. Along PC2, LGC, starch, β-sheet content, and FS were positively associated, while Td showed a negative association. Thus, the two principal components represented combinations of several measured characteristics rather than individual properties.

The score plot showed separation of the samples according to their overall physicochemical and functional profiles. The fine and split fractions occupied distinct regions of the PCA space, while the crushed samples were positioned separately from these groups. The separation of the crushed fraction indicates that it exhibited a distinct combination of the measured properties across PC1 and PC2. This position should therefore be interpreted as a multivariate characteristic of the crushed fraction rather than being attributed to a single property. The intermediate position of the crushed fraction between the fine and split fractions in the PCA space may reflect its intermediate particle size and the associated combination of composition, protein behaviour, hydration-related properties, and structural characteristics.

Interestingly, the fine fraction did not cluster with the crushed fraction despite having the highest apparent protein content. This observation highlights that protein concentration alone is not a reliable indicator of ingredient functionality. Instead, functional performance depends on the interaction between protein accessibility, molecular conformation, fibre composition, and the ability of components to form organised structures [67]. The PCA therefore supports the conclusion that excessive particle size reduction may increase substrate accessibility but does not necessarily produce superior functional ingredients. However, PCA represents relationships among measured variables and does not establish direct causality between individual properties.

Pearson correlation analysis (Supplementary Materials) further supported the relationships among key functional parameters. The positive association between protein solubility and emulsifying activity and foaming capacity indicates that protein dispersibility is a major determinant of interfacial performance. This agrees with previous studies showing that soluble and flexible proteins are more capable of migrating to interfaces and forming stabilising films [68]. The association between structural parameters and functionality also suggests that controlled molecular rearrangement contributes to improved ingredient performance. Changes in protein secondary structure, thermal behaviour, and fibre composition were associated with functional improvements, supporting the concept that fermentation modifies the lupin matrix through coordinated changes rather than isolated effects [69]. Overall, PCA and correlation analysis provide additional evidence that the crushed fraction represents an optimal balance between fermentation-induced modification and preservation of functional structure. These analyses reinforce the importance of considering multiple quality attributes when designing fermented plant-based ingredients, as improvements in one parameter, such as protein concentration, may not necessarily translate into enhanced overall functionality.

## 5. Conclusions

This study demonstrated that particle size control before fermentation is an effective approach to tailor the physicochemical, structural, and functional properties of lupin flour fermented with *R. oligosporus*. The effect of particle size was not linear, with the crushed fraction achieving the most favourable balance between fungal accessibility and preservation of structural integrity. Compared with split and fine fractions, fermented crushed lupin exhibited improved protein solubility, water holding capacity, emulsifying and foaming properties, and rheological strength. Although the fine fraction showed the highest apparent protein content, its lower functional performance indicated that protein concentration alone does not determine ingredient functionality. Fermentation induced controlled modification of the lupin matrix through protein restructuring, lipid utilisation, and dietary fibre transformation, while maintaining overall protein stability. Multivariate analysis further confirmed that functional performance was governed by the interaction of compositional, structural, and molecular characteristics rather than by a single quality parameter.

Nevertheless, the present study focused primarily on the physicochemical, structural and functional characteristics of the fermented lupin flours, and direct measurements of fungal biomass development, substrate utilisation, enzyme activity, oxygen transfer and detailed fermentation kinetics were not conducted. Future studies should incorporate these parameters to provide a more comprehensive understanding of the relationship between substrate particle size and *R. oligosporus* fermentation behaviour and to further elucidate the mechanisms underlying the observed changes in fermented lupin flour properties. Further evaluation using model and real food systems is also required to establish the application-specific performance of the fermented flours, including their processing, storage and sensory characteristics.

## Figures and Tables

**Table 1:** Mean values and standard deviations of d_F_ and min d_F_ for lupin samples.

Fermentation Stage	Fraction	Feret Diameter, d_F_ (mm)	Minimum Feret Diameter, min d_F_ (mm)
Start of fermentation	Split	7.02 ± 1.60 ^a^	6.21 ± 1.04 ^a^
	Crushed	3.72 ± 0.56 ^b^	2.92 ± 0.92 ^b^
	Fine	0.37 ± 0.25 ^c^	0.15 ± 0.07 ^c^
End of fermentation	Split	8.50 ± 1.72 ^a^	7.20 ± 2.84 ^a^
	Crushed	5.10 ± 1.21 ^b^	4.41 ± 1.02 ^b^
	Fine	0.61 ± 0.09 ^c^	0.27 ± 0.07 ^c^

Note: Values are presented as mean ± standard deviation. Different superscript letters within each parameter indicate significant differences according to Tukey’s HSD test at *p* < 0.05.

**Table 2:** Proximate composition of freeze-dried unfermented and fermented lupin flours.

Sample	Ash (%)	Crude Protein (%)	Fat (%)	IDF (%)	SDF (%)	Starch (%)	Carbohydrates, by Difference (%)
Unfermented	2.9 ± 0.1 ^a^	37.1 ± 0.5 ^ab^	6.5 ± 0.3 ^a^	35.6 ± 1.2 ^a^	10.3 ± 0.6 ^c^	0.4 ± 0.05 ^a^	48.7 ± 0.8 ^b^
Fermented Split	3.1 ± 0.1 ^a^	32.2 ± 0.6 ^b^	4.7 ± 0.3 ^b^	20.03 ± 2.9 ^b^	6.93 ± 4.2 ^c^	0.35 ± 0.04 ^a^	55.2 ± 1.2 ^a^
Fermented Crushed	3.3 ± 0.1 ^a^	39.5 ± 0.7 ^ab^	2.7 ± 0.2 ^c^	10.6 ± 0.8 ^c^	28.9 ± 1.5 ^a^	0.33 ± 0.04 ^a^	50.2 ± 1.0 ^ab^
Fermented Fine	3.5 ± 0.1 ^a^	43.5 ± 0.8 ^a^	2.0 ± 0.2 ^c^	16.23 ± 1.0 ^c^	13.7 ± 0.6 ^b^	0.24 ± 0.03 ^b^	47.0 ± 1.0 ^b^

Note: Data represent the mean and standard deviation. Different lowercase letters in the same column indicate significant differences (*p* < 0.05). IDF: Insoluble Dietaty Fibre, SDF: Soluble Dietary Fibre. Carbohydrates calculated by difference.

**Table 3:** Amino acid profile of unfermented and fermented flour fractions.

Amino Acids (mg/100 mg)	Samples			
	Unfermented	Fermented Split	Fermented Crushed	Fermented Fine
Essential Amino Acids (EAA)				
Phenylalanine	1.66 ± 0.11 ^b^	1.57 ± 0.02 ^c^	1.66 ± 0.02 ^b^	1.78 ± 0.01 ^a^
Histidine	0.70 ± 0.01 ^a^	0.62 ± 0.01 ^a^	0.64 ± 0.14 ^a^	0.65 ± 0.21 ^a^
Isoleucine	1.64 ± 0.02 ^a^	1.55 ± 0.02 ^b^	1.69 ± 0.02 ^a^	1.60 ± 0.02 ^ab^
Leucine	2.56 ± 0.32 ^a^	2.48 ± 0.11 ^b^	2.58 ± 0.14 ^a^	2.64 ± 0.17 ^a^
Lysine	1.95 ± 0.02 ^a^	1.74 ± 0.02 ^b^	1.84 ± 0.02 ^ab^	1.83 ± 0.02 ^ab^
Methionine	0.30 ± 0.02 ^b^	0.30 ± 0.02 ^b^	0.42 ± 0.02 ^a^	0.45 ± 0.02 ^a^
Threonine	1.37 ± 0.05 ^a^	1.29 ± 0.13 ^a^	1.33 ± 0.12 ^a^	1.17 ± 0.11 ^b^
Valine	1.68 ± 0.13 ^ab^	1.60 ± 0.24 ^b^	1.77 ± 0.34 ^a^	1.88 ± 0.02 ^a^
Total EAA	11.86 ± 0.68 ^ab^	11.15 ± 0.57 ^c^	12.33 ± 0.96 ^a^	12.00 ± 0.58 ^a^
Non-essential Amino Acids (NEAA)				
Aspartic Acid	3.97 ± 0.02 ^a^	3.70 ± 0.11 ^ab^	3.73 ± 0.12 ^ab^	3.12 ± 0.04 ^b^
Serine	2.08 ± 0.13 ^a^	1.92 ± 0.01 ^a^	1.75 ± 0.02 ^ab^	1.41 ± 0.21 ^b^
Glutamic Acid	10.72 ± 0.03 ^a^	7.94 ± 0.02 ^ab^	7.36 ± 0.02 ^b^	10.68 ± 0.11 ^a^
Proline	1.84 ± 0.03 ^a^	1.60 ± 0.02 ^b^	1.69 ± 0.02 ^ab^	1.63 ± 0.04 ^ab^
Glycine	1.60 ± 0.04 ^c^	1.82 ± 0.11 ^ab^	1.75 ± 0.12 ^b^	2.08 ± 0.03 ^a^
Alanine	1.36 ± 0.12 ^b^	1.28 ± 0.02 ^b^	1.87 ± 0.04 ^a^	1.99 ± 0.02 ^a^
Tyrosine	1.92 ± 0.12 ^a^	1.74 ± 0.22 ^a^	1.86 ± 0.12 ^ab^	2.00 ± 0.02 ^a^
Arginine	4.45 ± 0.10 ^a^	3.88 ± 0.14 ^ab^	2.59 ± 0.12 ^b^	1.84 ± 0.11 ^c^
Cysteine	0.35 ± 0.04 ^ab^	0.32 ± 0.03 ^b^	0.33 ± 0.11 ^b^	0.40 ± 0.14 ^a^
Total NAA	28.29 ± 0.63 ^a^	23.20 ± 0.88 ^b^	22.93 ± 0.83 ^b^	25.15 ± 0.68 ^a^

Note: Data represent the mean and standard deviation. Different lowercase letters in the same column indicate significant differences (*p* < 0.05).

**Table 4:** Colour parameters of unfermented and fermented lupin fractions.

Sample	Colour			∆E
	L*	a*	b*	
Unfermented	63.71 ± 5.42 ^b^	6.43 ± 1.57 ^a^	36.53 ± 8.33 ^a^	
Fermented Split	64.57 ± 6.28 ^b^	6.11 ± 1.52 ^a^	36.01 ± 8.72 ^a^	1.38 ± 0.57 ^b^
Fermented Crushed	65.98 ± 7.32 ^a^	6.16 ± 1.23 ^a^	35.71 ± 7.62 ^a^	2.75 ± 0.72 ^a^
Fermented Fine	63.58 ± 9.52 ^b^	6.32 ± 1.28 ^a^	36.32 ± 8.26 ^a^	0.63 ± 0.43 ^c^

Note: ΔE was calculated relative to the mean colour values of the unfermented lupin flour using the CIELAB colour-difference equation. Values are presented as mean ± standard deviation (*n* = 3). Different lowercase letters in the same column indicate significant differences (*p* < 0.05).

**Table 5:** Functional properties of lupin flour and their fermented counterparts.

Sample	Water Holding Capacity (WHC) (%)	Oil Holding Capacity (OHC) (%)	Least Gelation Concentration (LGC) (%)
Unfermented	223 ± 8.62 ^b^	209 ± 6.52 ^a^	14 ^a^
Fermented Split	238 ± 6.58 ^b^	196 ± 8.80 ^b^	12 ^b^
Fermented Crushed	258 ± 8.11 ^a^	207 ± 6.25 ^ab^	13 ^ab^
Fermented Fine	236 ± 6.52 ^b^	205 ± 6.55 ^ab^	13 ^ab^

Note: Data represent the mean and standard deviation. Different lowercase letters in the same column indicate significant differences (*p* < 0.05).

**Table 6:** Functional properties of lupin flour and their fermented counterparts across different pH ranges.

Functional Tests	pH			
	2	4	6	8
Emulsifying Activity (%)				
Unfermented	44.60 ± 10.21 ^c^	40.2 ± 10.14 ^c^	45.7 ± 10.10 5 ^c^	46.3 ± 7.21 ^c^
Fermented Split	46.10 ± 10.11 ^b^	41.20 ± 8.72 ^b^	47.20 ± 8.14 ^b^	47.80 ± 8.12 ^b^
Fermented Crushed	47.60 ± 9.42 ^a^	43.20 ± 7.11 ^a^	48.20 ± 9.71 ^a^	48.80 ± 8.24 ^a^
Fermented Fine	42.60 ± 8.74 ^d^	40.20 ± 8.54 ^d^	45.70 ± 7.24 ^c^	46.30 ± 7.15 ^c^
Emulsifying Stability (%)				
Unfermented	38.30 ± 10.44 ^c^	36.5 ± 7.42 ^c^	44.5 ± 7.12 ^c^	45.4 ± 0.4 ^c^
Fermented Split	41.30 ± 40.72 ^a^	39.00 ± 8.11 ^a^	47.00 ± 8.81 ^a^	47.40 ± 1.34 ^a^
Fermented Crushed	36.30 ± 8.24 ^d^	34.00 ± 8.24 ^d^	41.50 ± 7.24 ^d^	42.40 ± 3.22 ^d^
Fermented Fine	39.30 ± 8.22 ^b^	37.00 ± 6.21 ^b^	46.00 ± 7.67 ^b^	46.40 ± 4.11 ^b^
Foaming Capacity (%)				
Unfermented	32.10 ± 9.41 ^c^	20.6 ± 7.71 ^c^	45.70 ± 8.22 ^c^	50.70 ± 7.21 ^c^
Fermented Split	33.60 ± 8.22 ^b^	21.60 ± 5.24 ^b^	47.70 ± 7.21 ^b^	53.20 ± 8.21 ^b^
Fermented Crushed	35.10 ± 8.87 ^a^	23.10 ± 4.46 ^a^	50.00 ± 8.61 ^a^	55.70 ± 7.65 ^a^
Fermented Fine	30.60 ± 9.21 ^d^	19.60 ± 5.42 ^d^	43.70 ± 7.64 ^c^	49.20 ± 8.34 ^c^
Foaming Stability (%)				
Unfermented	22.70 ± 5.21 ^c^	14.5 ± 4.71 ^c^	40.60 ± 6.72 ^c^	45.70 ± 10.24 ^c^
Fermented Split	24.52 ± 4.22 ^a^	15.66 ± 3.12 ^a^	43.85 ± 8.21 ^a^	49.36 ± 9.24 ^a^
Fermented Crushed	20.88 ± 4.24 ^d^	13.34 ± 2.14 ^d^	37.35 ± 8.40 ^d^	42.04 ± 8.11 ^d^
Fermented Fine	22.02 ± 6.44 ^b^	14.07 ± 2.71 ^b^	39.38 ± 10.47 ^b^	44.33 ± 8.12 ^b^
Protein solubility (%)				
Unfermented	30.57 ± 7.11 ^bc^	22.50 ± 5.61 ^e^	58.20 ± 11.24 ^a^	61.10 ± 9.21 ^a^
Fermented Split	31.80 ± 8.12 ^b^	23.60 ± 5.21 ^d^	60.50 ± 8.14 ^b^	62.90 ± 8.80 ^b^
Fermented Crushed	33.50 ± 9.22 ^a^	24.80 ± 4.31 ^f^	62.50 ± 8.62 ^c^	64.80 ± 11.61 ^c^
Fermented Fine	29.00 ± 7.24 ^c^	21.80 ± 4.92 ^de^	56.50 ± 10.61 ^c^	59.00 ± 10.24 ^bc^

Note: Data represent the mean and standard deviation. Different lowercase letters in the same column indicate significant differences (*p* < 0.05).

**Table 7:** Protein secondary structure of unfermented and fermented lupin.

Secondary Structure	Unfermented (%)	Fermented Split (%)	Fermented Crushed (%)	Fermented Fine (%)
β-sheet	36.25 ± 3.47 ^a^	34.35 ± 4.11 ^a^	35.92 ± 5.11 ^a^	35.13 ± 4.33 ^a^
Random coil	15.25 ± 4.11 ^a^	13.81 ± 2.71 ^b^	13.48 ± 2.34 ^b^	13.52 ± 3.14 ^b^
α-helix	16.11 ± 4.33 ^b^	15.34 ± 2.07 ^b^	19.78 ± 4.44 ^a^	19.44 ± 2.14 ^a^
β-turn	29.26 ± 2.76 ^a^	30.74 ± 4.31 ^a^	27.77 ± 4.17 ^b^	27.35 ± 5.21 ^b^
Aggregated strands	2.86 ± 0.37 ^b^	5.76 ± 1.11 ^a^	3.04 ± 1.47 ^ab^	4.56 ± 0.78 ^a^

Note: Values are presented as mean ± SD (*n* = 3). Different superscript letters within the same row indicate significant differences among treatments (*p* < 0.05).

**Table 8:** Denaturation temperatures and enthalpy changes across all samples.

Sample	Peak 1		Peak 2	
	Denaturation Temperature (T_d_)	Enthalpy Change (ΔH) J/g	Denaturation Temperature (T_d_)	Enthalpy Change (ΔH) J/g
Unfermented	88.40 ± 10.02 ^a^	3.30 ± 1.44 ^b^	102.41 ± 10.24 ^a^	2.62 ± 1.05 ^c^
Fermented Split	88.92 ± 8.21 ^a^	5.10 ± 1.72 ^a^	104.92 ± 9.14 ^a^	3.11 ± 1.12 ^b^
Fermented Crushed	89.51 ± 8.43 ^a^	6.72 ± 2.11 ^a^	104.56 ± 9.11 ^a^	4.71 ± 1.41 ^a^
Fermented Fine	88.73 ± 8.712 ^a^	2.32 ± 1.08 ^b^	105.47 ± 7.20 ^a^	1.77 ± 0.52 ^d^

Note: data represent the mean and standard deviation. Different lowercase letters in the same category indicate significant differences (*p* ≤ 0.05).

## Data Availability

Data will be made available upon request.

## Supplementary Materials

The following supporting information can be downloaded at: https://www.mdpi.com/article/10.3390/foods15183306/s1, Table S1: Pearson correlations analysis of fermented lupin flours; Figure S1: Strain sweeps of lupin flours; Table S2: Dry matter content of lupin flours; Table S3: Dry matter recovery of lupin flours.
