# Supplementary material for: Effect of Particle Size on the Physicochemical and Functional Properties of Sweet Lupin (Lupinus angustifolius) Fermented with Rhizopus oligosporus
Source: Foods. 2026 Sep 18;15(18):3306. doi: 10.3390/foods15183306 (PMC13606211; doi:10.3390/foods15183306)
Supplement: Supplementary file 1 [file foods-15-03306-s001.zip › foods-4502247-supplementary.pdf]

Supplementary Materials

Table S1. Pearson correlations analysis of fermented lupin flours

Pearson correlations analysis of fermented lupin flours

	Protein	EA	ES	FA	FS	WHC	OHC	LGC	Solubility	Moisture	Ash	Fat	IDF	SDF	Starch	Carbohyd	Phenylala	Histidine	Isoleucin	Leucine	Lysine	Methionir	Threonine	Valine	Aspartic_	Serine	Glutamic_	Proline	Glycine	Alanine	Tyrosine	Arginine	Cysteine	L	a	b	Aggregate	B_sheets	Random_	A_helix	B_turn	Td_1	Td	Enthaply	Enthalpy_
Protein	1																																												
EA	-0.321																																												
ES	-0.335	-0.572																																											
FA	-0.402	.978**	-.621*																																										
FS	-0.543	0.227	0.042	0.303																																									
WHC	-.933**	0.256	0.456	0.304	0.518																																								
OHC	0.353	-0.129	-.670*	0.014	-0.033	-0.47																																							
LGC	0.432	-0.493	-0.377	-0.358	-0.111	-0.525	.912**																																						
Solubility	-0.488	.942**	-.597*	.990**	0.364	0.375	0.064	-0.299																																					
Moisture	-.992**	0.286	0.335	0.376	.585*	.952**	-0.313	-0.383	0.468																																				
Ash	.687*	0.09	-0.075	-0.1	-0.425	-0.54	-0.342	-0.352	-0.236	-.716**																																			
Fat	-.655*	-0.273	0.252	-0.093	0.451	0.562	0.279	0.36	0.046	.698*	-.970**																																		
IDF	-0.309	-.651*	0.353	-0.489	0.23	0.249	0.382	.593*	-0.364	0.36	-.808**	.900**																																	
SDF	0.46	.638*	-.941**	.617*	-0.159	-0.533	0.437	0.143	0.548	-0.477	0.39	-0.558	-.639*																																
Starch	-.614*	0.149	-0.18	0.332	.577*	0.517	0.419	0.332	0.451	.664*	-.874**	.838**	.613*	-0.117																															
Carbohydrates	-.882**	.586*	0.269	.581*	0.469	.878**	-.616*	-.775**	.607*	.856**	-0.281	0.233	-0.155	-0.244	0.303																														
Phenylalanine	.949**	-0.485	-0.048	-.593*	-0.565	-.839**	0.138	0.301	-.681*	-.945**	.730**	-.646*	-0.258	0.211	-.735**	-.826**																													
Histidine	0.157	-0.454	-0.257	-0.293	0.019	-0.247	.840**	.922**	-0.204	-0.104	-0.574	.589*	.739**	-0.028	0.538	-0.559	0.034																												
Isoleucine	0.458	0.287	-.934**	0.364	-0.054	-0.549	.847**	.637*	0.358	-0.432	-0.012	-0.12	-0.111	.819**	0.204	-0.504	0.188	0.508																											
Leucine	.925**	-0.501	-0.146	-0.569	-0.5	-.892**	0.291	0.466	-.635*	-.921**	.583*	-0.509	-0.127	0.248	-0.548	-.880**	.927**	0.176	0.287																										
Lysine	0.311	-0.424	-0.396	-0.27	-0.032	-0.42	.936**	.985**	-0.198	-0.263	-0.457	0.455	.635*	0.133	0.443	-.674*	0.166	.954**	.643*	0.339																									
Methionine	.868**	0.059	-0.352	-0.089	-0.508	-.771**	0.014	-0.019	-0.215	-.888**	.919**	-.925**	-.716**	.595*	-.793**	-0.546	.826**	-0.266	0.336	.727**	-0.121																								
Threonine	-0.519	0.299	-0.435	0.489	0.463	0.35	.576*	0.41	.597*	0.552	-.846**	.735**	0.492	0.135	.923**	0.228	-.706*	0.557	0.448	-0.515	0.523	-.673*																							
Valine	.973**	-0.224	-0.286	-0.341	-0.564	-.903**	0.187	0.237	-0.447	-.985**	.817**	-.791**	-0.474	0.474	-.756**	-.768**	.946**	-0.043	0.354	.883**	0.119	.940**	-.650*																						
Aspartic_Acid	-.649*	0.277	-0.293	0.465	0.522	0.491	0.465	0.311	.582*	.678*	-.904**	.807**	0.541	-0.007	.936**	0.347	-.797**	0.513	0.304	-.644*	0.438	-.775**	.979**	-.764**																					
Serine	-.780**	0.104	0.006	0.286	0.534	.646*	0.277	0.216	0.417	.809**	-.972**	.927**	.682*	-0.302	.929**	0.439	-.843**	0.458	0.022	-.696*	0.34	-.923**	.892**	-.887**	.952**																				
Glutamic_Acid	0.462	-.978**	0.391	-.947**	-0.277	-0.415	0.301	.639*	-.916**	-0.423	-0.058	0.221	.623*	-0.473	-0.143	-.734**	0.575	0.565	-0.088	.614*	0.564	0.047	-0.248	0.342	-0.261	-0.142																			
Proline	0.042	-0.36	-0.316	-0.173	0.138	-0.167	.871**	.916**	-0.072	0.014	-.685*	.674*	.765**	0.003	.658*	-0.461	-0.096	.941**	0.545	0.106	.953**	-0.39	.710**	-0.165	.647*	.586*	0.466																		
Glycine	0.534	-0.207	0.361	-0.404	-0.448	-0.362	-.580*	-0.448	-0.52	-0.566	.896**	-.798**	-.580*	-0.046	-.928**	-0.202	.697*	-.613*	-0.402	0.516	-0.561	.718**	-.992**	.672*	-.986**	-.922**	0.164	-.748**																	
Alanine	.903**	0.07	-0.467	-0.056	-0.523	-.850**	0.15	0.099	-0.176	-.925**	.862**	-.892**	-.679*	.679*	-.721**	-.616*	.825**	-0.18	0.448	.762**	-0.006	.978**	-0.572	.955**	-.700*	-.877**	0.059	-0.273	.624*																
Tyrosine	.927**	-.627*	-0.141	-.660*	-0.5	-.872**	0.453	.639*	-.705*	-.903**	0.439	-0.357	0.059	0.179	-0.458	-.972**	.913**	0.408	0.363	.934**	0.526	.641*	-0.418	.846**	-0.528	-.589*	.751**	0.289	0.397	.682*															
Arginine	-.787**	-0.104	0.242	0.068	0.51	.688*	0.167	0.197	0.203	.821**	-.974**	.980**	.798**	-0.529	.860**	0.414	-.782**	0.451	-0.17	-.659*	0.307	-.971**	.765**	-.895**	.851**	.967**	0.033	0.556	-.816**	-.948**	-0.535														
Cysteine	.808**	-.756**	0.251	-.841**	-0.521	-.702*	0.073	0.351	-.896**	-.799**	0.535	-0.393	0.041	-0.137	-.650*	-.805**	.921**	0.169	-0.046	.868**	0.229	.604*	-.698*	.785**	-.743**	-.693*	.802**	0.002	.652*	0.576	.902**	-0.554													
L	-0.52	.964**	-0.344	.939**	0.317	0.472	-0.325	-.655*	.915**	0.482	0.011	-0.166	-.579*	0.416	0.184	.776**	-.624*	-0.561	0.042	-.658*	-0.574	-0.109	0.271	-0.403	0.294	0.192	-.997**	-0.459	-0.191	-0.125	-.794**	0.025	-.831**												
a	0.404	-.990**	0.54	-.984**	-0.232	-0.324	0.125	0.484	-.961**	-0.369	0.01	0.181	.576*	-.580*	-0.202	-.633*	0.564	0.421	-0.263	.583*	0.406	0.037	-0.366	0.313	-0.355	-0.195	.978**	0.316	0.281	0.025	.686*	0.008	.812**	-.968**											
b	0.409	-.990**	0.503	-.975**	-0.25	-0.338	0.176	0.533	-.947**	-0.373	-0.029	0.21	.605*	-0.561	-0.184	-.660*	0.563	0.483	-0.21	0.57	0.458	0.031	-0.325	0.308	-0.314	-0.164	.988**	0.367	0.233	0.018	.696*	0.032	.809**	-.980**	.992**										
Aggregate_Strands	0	-0.018	.623*	-0.202	-0.17	0.164	-.922**	-.811**	-0.287	-0.041	.633*	-0.538	-0.506	-0.327	-.697*	0.324	0.224	-.826**	-.753**	0.032	-.870**	0.315	-.839**	0.177	-.754**	-.597*	-0.118	-.918**	.841**	0.173	-0.125	-0.473	0.254	0.123	0.057	0.001									
B_sheets	-0.132	-.892**	.703*	-.816**	0.046	0.165	0.042	0.38	-.731**	0.173	-0.468	.640*	.868**	-.856**	0.202	-0.226	0.044	0.476	-0.448	0.088	0.369	-0.492	0.004	-0.24	0.079	0.308	.815**	0.434	-0.106	-0.513	0.232	0.519	0.391	-.773**	.841**	.845**	-0.062								
Random_coils	-0.087	-.709**	0.218	-0.552	0.11	-0.035	0.533	.740**	-0.442	0.12	-.693*	.766**	.942**	-0.491	0.474	-0.37	-0.065	.797**	0.034	0.093	.764**	-0.543	0.426	-0.245	0.438	0.535	.721**	.824**	-0.51	-0.466	0.27	.634*	0.199	-.692*	.647*	.675*	-.578*	.819**							
A_helix	-.582*	-.577*	.814**	-0.501	0.299	.609*	-0.252	0	-0.401	.610*	-.623*	.766**	.790**	-.960**	0.378	0.279	-0.386	0.211	-.679*	-0.369	0.041	-.773**	0.143	-.636*	0.277	0.545	0.43	0.216	-0.231	-.827**	-0.267	.738**	-0.033	-0.367	0.503	0.496	0.078	.871**	.630*						
B_turn	0.188	.826**	-.914**	.818**	0.008	-0.275	0.324	-0.029	.764**	-0.209	0.25	-0.443	-.648*	.956**	0.033	0.037	-0.065	-0.13	.733**	-0.033	-0.004	0.404	0.276	0.222	0.166	-0.117	-.695*	-0.064	-0.179	0.475	-0.113	-0.362	-0.41	.650*	-.786**	-.769**	-0.311	-.926**	-0.563	-.885**					
Td_1	0.032	.929**	-.653*	.856**	0.012	-0.061	-0.109	-0.453	.776**	-0.073	0.423	-.594*	-.858**	.804**	-0.157	0.329	-0.132	-0.521	0.381	-0.181	-0.431	0.417	0.024	0.148	-0.038	-0.25	-.871**	-0.47	0.079	0.429	-0.33	-0.457	-0.468	.835**	-.885**	-.891**	0.096	-.994**	-.837**	-.814**	.902**				
Td	0.251	0.28	0.184	0.076	-0.283	-0.112	-.732**	-.764**	-0.048	-0.302	.867**	-.852**	-.847**	0.157	-.811**	0.196	0.361	-.877**	-0.375	0.168	-.833**	.636*	-.824**	0.444	-.807**	-.793**	-0.339	-.955**	.871**	0.536	-0.017	-.773**	0.215	0.314	-0.212	-0.262	.879**	-0.484	-.836**	-0.401	0.145	0.49			
Enthaply	-0.413	.981**	-.606*	1.000**	0.306	0.317	-0.007	-0.377	.989**	0.386	-0.095	-0.096	-0.495	.605*	0.326	.597*	-.598*	-0.309	0.344	-.578*	-0.289	-0.093	0.478	-0.348	0.458	0.284	-.953**	-0.191	-0.393	-0.063	-.672*	0.067	-.844**	.946**	-.986**	-.979**	-0.183	-.815**	-0.562	-0.493	.810**	.856**	0.09		
Enthalpy_2	-0.272	.935**	-.767**	.977**	0.261	0.152	0.216	-0.162	.971**	0.253	-0.119	-0.085	-0.44	.728**	0.369	0.409	-0.506	-0.129	0.548	-0.45	-0.08	-0.03	0.562	-0.244	0.508	0.287	-.862**	-0.01	-0.476	0.031	-0.516	0.047	-.774**	.845**	-.936**	-.917**	-0.371	-.818**	-0.461	-.586*	.891**	.838**	-0.042	.972**	1

Figure S1. Strain sweeps of lupin flours

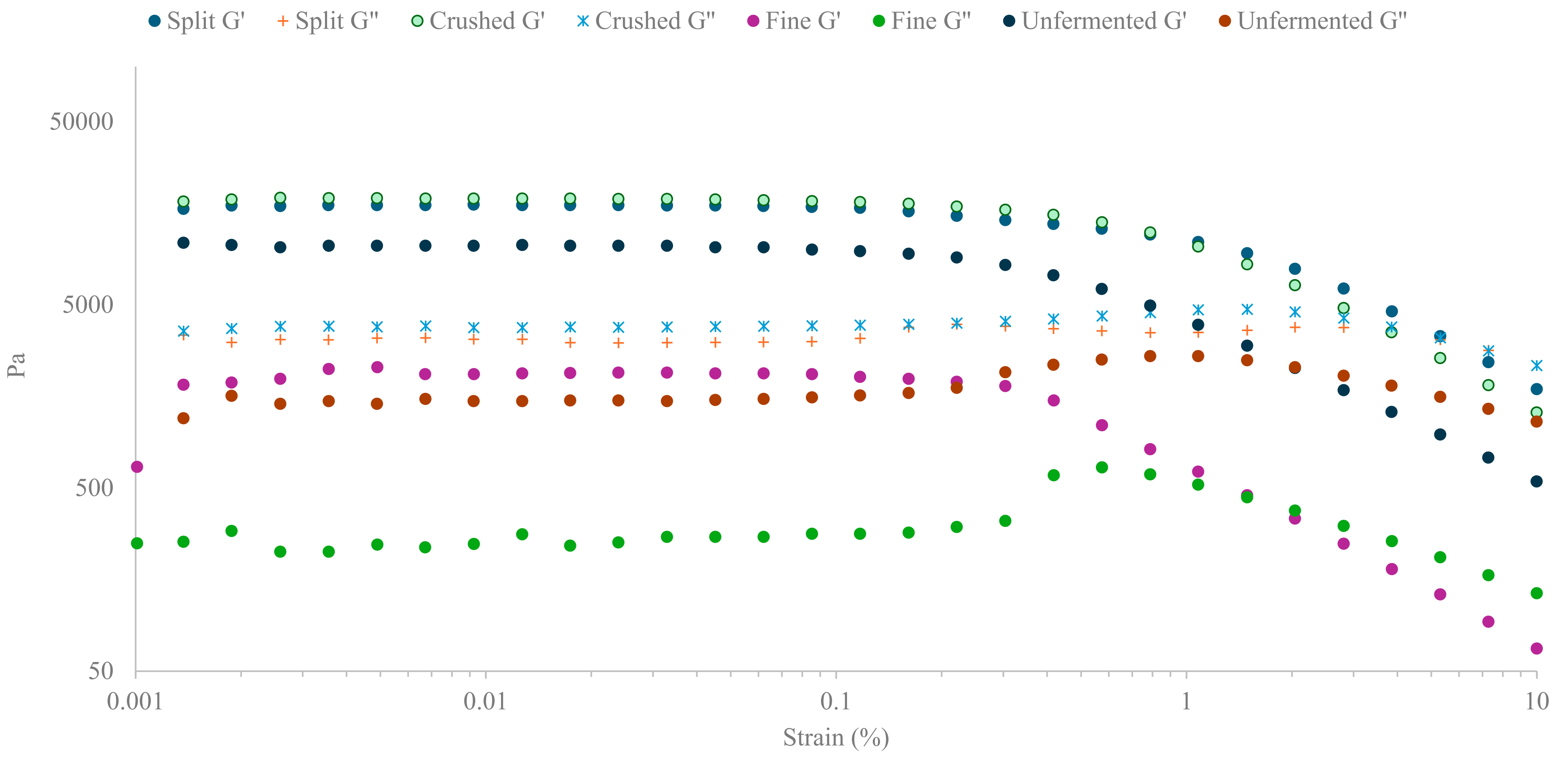

Table S2. Dry matter content of lupin flours

Particle size fraction	Sampling time point (h)				DM mean (%)
	0	12	24	36	
Split	24.01 ± 0.55	24.90 ± 1.43	24.48 ± 0.80	23.61 ± 0.91	**24.25 ± 0.15[a]**
Crushed	24.24 ± 0.30	23.93 ± 0.25	22.58 ± 1.62	23.61 ± 1.26	**23.59 ± 0.53[ab]**
Fine	23.22 ± 0.49	23.50 ± 0.28	22.84 ± 0.40	21.88 ± 1.41	**22.86 ± 0.20[b]**

Values are mean ± SD (n = 3). Different superscript letters indicate significant differences among particle-size fractions ($p < 0.05$), based on Bonferroni-adjusted pairwise comparisons.

Table S3. Dry Matter Recovery of lupin flours

Particle-size fraction	DM recovery (%)
Split	97.20 ± 0.84[a]
Crushed	95.52 ± 1.30[a]
Fine	94.53 ± 1.37[a]
**Overall**	**95.75 ± 1.56**

Values are presented as mean ± standard deviation (n = 3). Means within the same column sharing the same superscript letter are not significantly different according to Tukey's HSD test ($p > 0.05$).
